# Incongruence between transcriptional and vascular pathophysiological cell states

**DOI:** 10.1038/s44161-023-00272-4

**Published:** 2023-05-29

**Authors:** Macarena Fernández-Chacón, Severin Mühleder, Alvaro Regano, Lourdes Garcia-Ortega, Susana F. Rocha, Carlos Torroja, Maria S. Sanchez-Muñoz, Mariya Lytvyn, Verónica Casquero-Garcia, Macarena De Andrés-Laguillo, Lars Muhl, Michael M. Orlich, Konstantin Gaengel, Emilio Camafeita, Jesús Vázquez, Alberto Benguría, M. Luisa Iruela-Arispe, Ana Dopazo, Fátima Sánchez-Cabo, Hannah Carter, Rui Benedito

**Affiliations:** 1 Molecular Genetics of Angiogenesis Group, Centro Nacional de Investigaciones Cardiovasculares (CNIC), Madrid, Spain; 2 Faculty of Health Sciences, Universidad Loyola Andalucía, Seville, Spain; 3 Bioinformatics Unit, Centro Nacional de Investigaciones Cardiovasculares (CNIC), Madrid, Spain; 4 Department of Medicine, Huddinge, Karolinska Institutet, Huddinge, Sweden; 5 Department of Immunology, Genetics and Pathology, Rudbeck Laboratory, Uppsala, Sweden; 6 Cardiovascular Proteomics Laboratory, Centro Nacional de Investigaciones Cardiovasculares (CNIC), Madrid, Spain; 7 CIBER de Enfermedades Cardiovasculares (CIBERCV), Madrid, Spain; 8 Genomics Unit, Centro Nacional de Investigaciones Cardiovasculares (CNIC), Madrid, Spain; 9 Department of Cell and Development Biology, Feinberg School of Medicine, Northwestern University, Chicago, IL, USA; 10 Division of Medical Genetics, Department of Medicine and Moores Cancer Center, University of California San Diego, La Jolla, CA, USA

## Abstract

The Notch pathway is a major regulator of endothelial transcriptional specification. Targeting the Notch receptors or Delta-like ligand 4 (Dll4) dysregulates angiogenesis. Here, by analyzing single and compound genetic mutants for all Notch signaling members, we find significant differences in the way ligands and receptors regulate liver vascular homeostasis. Loss of Notch receptors caused endothelial hypermitogenic cell-cycle arrest and senescence. Conversely, Dll4 loss triggered a strong Myc-driven transcriptional switch inducing endothelial proliferation and the tip-cell state. Myc loss suppressed the induction of angiogenesis in the absence of Dll4, without preventing the vascular enlargement and organ pathology. Similarly, inhibition of other pro-angiogenic pathways, including MAPK/ERK and mTOR, had no effect on the vascular expansion induced by Dll4 loss; however, anti-VEGFA treatment prevented it without fully suppressing the transcriptional and metabolic programs. This study shows incongruence between single-cell transcriptional states, vascular phenotypes and related pathophysiology. Our findings also suggest that the vascular structure abnormalization, rather than neoplasms, causes the reported anti-Dll4 antibody toxicity.

Notch is a cell-to-cell ligand-receptor signaling pathway that has a major influence on cell transcription and biology^1^, playing important roles in several diseases^2^. General Notch signaling or γ-secretase inhibitors have been used in clinics with undesired side effects, including disruption of the normal intestinal stem-cell differentiation^2,3^. Specific blocking antibodies are now available that target the various ligands and receptors of the Notch pathway^4–8^. Given the specificity of Dll4 expression in endothelial cells (ECs), targeting this ligand was initially thought to be an effective and safe strategy for specifically modulating Notch signaling and angiogenesis in disease, such as during tumor growth^6,7^. However, anti-Dll4 treatment was later shown to induce a loss of endothelial quiescence and vascular neoplasms, which were proposed to be the main cause of pathology in several organs^5,8,9^. This toxicity diminished the clinical appeal of Dll4/Notch blockers in cancer or cardiovascular disease settings.

Here, we characterized the effect of single or compound targeting of all Notch signaling members on adult mice vascular homeostasis. High-resolution single-cell RNA sequencing (scRNA-seq) and three-dimensional (3D) confocal microscopy of adult liver vessels revealed very significant differences in the way each Notch member regulates vascular signaling, structure and single-cell states. γ-Secretase inhibitors or removal of Notch receptors did not cause substantial vascular or organ disease. Abnormal proliferating and sprouting single-cell states were generated only after Dll4 targeting. Surprisingly, suppression of these angiogenic cell states by additional genetic or pharmacological targeting was insufficient to prevent vascular and organ disease. Conceptually, our data show that the major transcriptional changes and angiogenic cell states elicited by targeting Dll4 correlate with, but do not cause, the observed vascular pathophysiology. Instead, we propose that it is the unrelated vascular structure abnormalization and malfunction that leads to organ pathology and the reported toxicity of anti-Dll4 treatment^5,8^.

## Results

### Notch pathway expression and signaling in adult organ ECs

**Extended Data Fig. 1:**
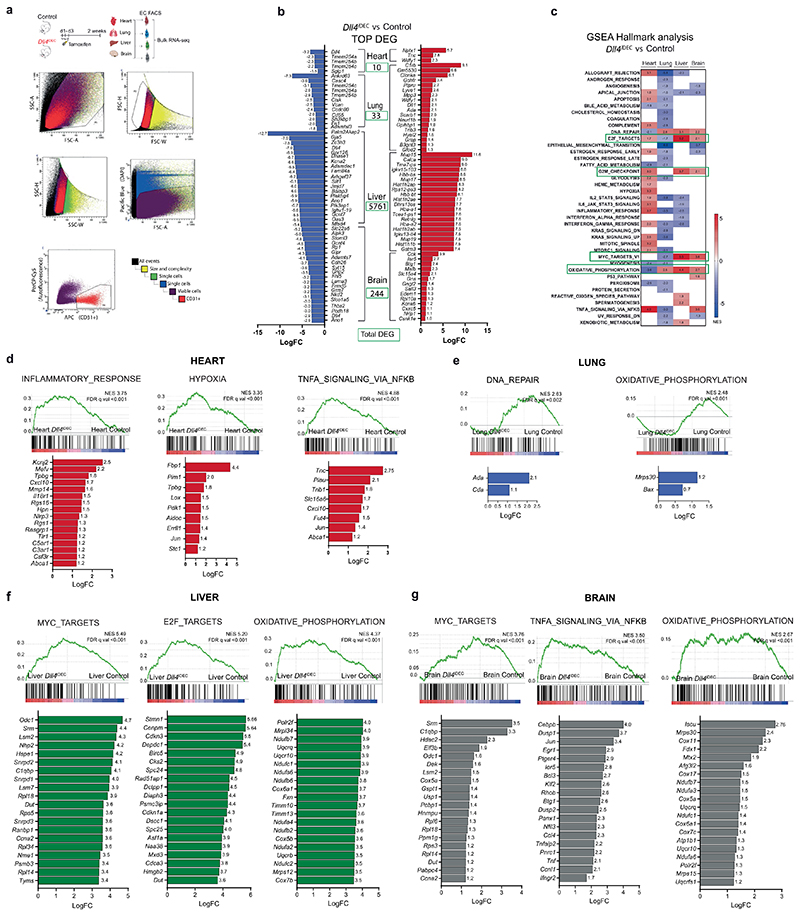
*Dll4* deletion elicits different gene expression signatures among different organ vascular beds. **a**, Schematic representation to illustrate the method used to obtain ECs for bulk RNA-seq analysis. FACS plots show the ECs gating strategy. The detectors, dyes and fluorophores are indicated in the X and Y-axes. **b**, List of the most up- and downregulated genes from the list of differentially expressed genes (DEG, absolute number boxed in green) based on the Benjamini and Hochberg adjusted p-value < 0.05. **c**, Heatmap with the normalized enrichment score (NES) from significant gene set enrichment (GSEA) hallmark analysis (FDR qval< 0.05). **d**, List of upregulated genes in *Dll4* mutant ECs within the top 3 enriched gene sets from GSEA hallmark analysis in heart. **e**, List of upregulated genes within the only enriched gene sets from GSEA Hallmark analysis in lung *Dll4^iDEC^* ECs compared to control ECs. **f**, List of upregulated genes within the top 3 enriched gene sets from GSEA hallmark analysis in Liver *Dll4^iDEC^* ECs compared to control ECs. **g**, List of upregulated genes within the top 3 enriched gene sets from GSEA Hallmark analysis in Brain *Dll4^iDEC^* ECs compared to control ECs. LogFC: Logarithmic Fold Change.

**Fig.1:**
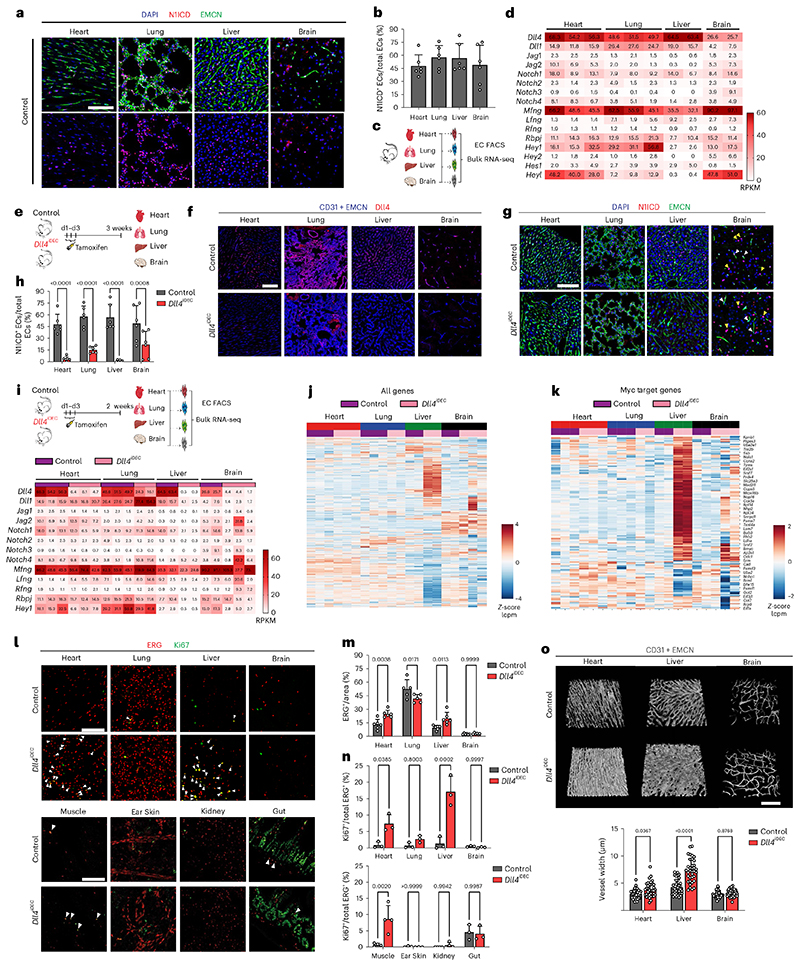
*Dll4* deletion leads to EC activation and proliferation only in some vascular beds. **a**,**b**, Notch1 signaling activity (cleaved Val1744 N1ICD) in quiescent endothelium (DAPI^+^Endomucin^+^, abbreviated as EMCN). **c**, Schematic representation to illustrate the bulk RNA-seq experiment performed with adult ECs isolated by FACS. **d**, Heatmap with RNA-seq reads per kilobase per million mapped reads (RPKM). **e**, Experimental layout for the inducible deletion of *Dll4* in Cdh5^+^ ECs *(Dll4^iDEC^)* with *Cdh5(PAC)-CreERT2.* **f**, Expression of Dll4 protein in CD31^+^EMCN^+^vessels. **g**,**h**, *Dll4* deletion significantly reduces Notch signaling activity (cleaved Val1744 N1ICD) in all quiescent vascular beds. In brain micrographs, white arrowheads indicate ECs and yellow arrowheads indicate non-ECs. Note that whereas N1ICD is maintained in non-ECs, most N1ICD signal disappears from the ECs in *Dll4^iDEC^* brains. **i**, Schematic representation to illustrate the bulk RNA-seq experiment performed with adult ECs. Below, a heatmap showing the relative expression of all Notch pathway components and canonical target genes in control and *Dll4^iDEC^* mutant ECs.**j**, Unsupervised hierarchical clustering showing stronger gene expression changes in *Dll4^iDEC^* liver ECs compared with the other organs. *Z*-score lcpm, *Z*-score of the logarithmic counts per million. **k**, Unsupervised hierarchical clustering showing strong upregulation of Myc target genes in *Dll4^iDEC^* liver ECs compared with the other organs. **l**-**n**, *Dll4* deletion results in increased EC proliferation (Ki67^+^ERG^+^ cells) in some organs but not others. **o**, 3D reconstruction images from thick vibratome sections show vessel (CD31^+^EMCN^+^) enlargement in *Dll4^iDEC^* heart and liver but not in brain. Data are presented as mean values ± s.d. For statistics, see Source Data File 1. Scale bars, 100 μm.

To elucidate the role of Notch signaling in global vascular homeostasis, we first assessed its activity in different organ vascular beds by immunodetection of the activated form of the Notch1 intracellular domain (N1ICD^Val1744^). This epitope was detected in ~50% of all organ ECs (Fig. 1a,b). Bulk RNA-seq analysis revealed that *Dll4* and *Notch1* are the most expressed ligand-receptor pair in quiescent vessels of most organs (Fig. 1c,d and Extended Data Fig. la), and that *Mfng* is the most str ongly expressed Notch glycosyltransferase. These enzymes are known to significantly enhance Delta ligand signaling and decrease Jagged ligand signaling^1^. Adult mice with induced deletion of *Dll4* in ECs *(Dll4^iDEC^* - *Dll4^flox/flox^Cdh5-CreERT2)* led to a significant reduction in N1ICD^Val1744^ and Hey1 signals in most organs’ quiescent ECs (Fig. 1e-i). This indicates that Dll4 is the main functional ligand responsible for triggering Notch activity in most quiescent vessels. We observed compensatory upregulation of *Dll1* only in lungs (Fig. 1i). *Dll4* deletion elicited remarkably different gene expression signatures among different organ vascular beds, with the adult liver endothelium presenting the most pronounced changes in gene expression (Fig. 1j,k and Extended Data Fig. 1). Despite significant transcriptional changes in most organs’ ECs, only the endothelium of the heart, muscle and liver showed an increase in the frequency of cycling or activated Ki67^+^ cells upon *Dll4* deletion (Fig. 1l-n), and these were the only organs with clear alterations in the 3D vascular architecture after the loss of Dll4-Notch signaling (Fig. 10). The brain underwent significant changes in gene expression (Fig. 1j,k and Extended Data Fig. 1), but these were not accompanied by endothelial proliferation or vascular morphological changes.

### Targeting Dll4 induces heterozonal responses in liver vessels

**Extended Data Fig. 2:**
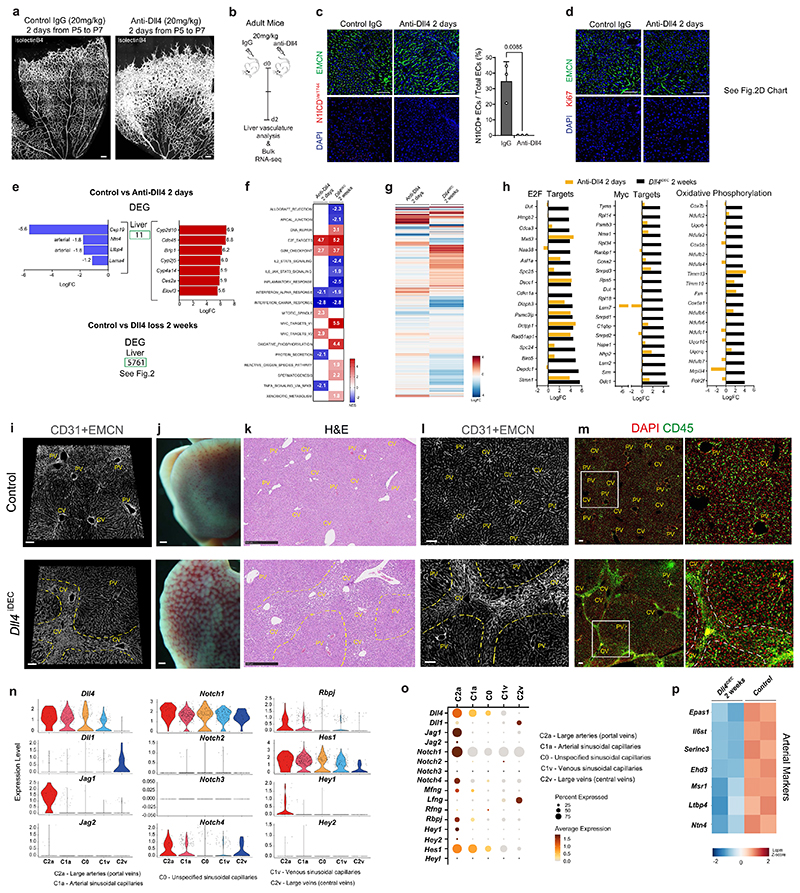
No major genetic and vascular changes after blocking Dll4 signaling in quiescent vessels for 2 days. **a**, Anti-Dll4 treatment for 48 h starting at postnatal day 5, results in a strong increase in retina vascular density and angiogenesis. **b**, Experimental layout for the antibody-based blockade of Dll4 ligand function in adult mice. **c-d**, Confocal micrographs showing that anti-Dll4 blockade for 2 days in adult mice significantly reduces Notch1 activity (cleaved Notch1Val1744), but not EC density (DAPI+ Endomucin+) and EC proliferation (Ki67+DAPI+ Endomucin+ cells) as depicted in chart D from Fig. 2. **e**, List of the few differential expressed genes (DEG) 2 days after Dll4 blockade in liver endothelium. **f**, Heatmap with the normalized enrichment score (NES) from significantly deregulated GSEA hallmark pathways (FDR qval< 0.05). **g**, Heatmap representing logFC of every expressed gene in the indicated conditions versus control livers. **h**, Comparison of gene expression fold changes between anti-Dll4 for 2 days (short-term) and *Dll4* deletion for 2 weeks (longterm). The top20 DEG belonging to the indicated GSEA pathways are shown. **i**, 3D projection images from confocal scanning of thick vibratome sections show that the vascular malformations observed in *Dll4^iDEC^* livers are located in sinusoids connecting central veins (CV), but not in sinusoids located close to portal veins (PVs). **j**, Low magnification stereomicroscope images of livers from control and *Dll4^iDEC^* mice showing liver pathology and blood accumulation in the mutants. **k**, Hematoxylin and Eosin staining images of liver sections from control and *Dll4^iDEC^* mice show sinusoidal dilation in areas surrounding and connecting central veins (CVs). **l**, Confocal micrographs showing higher EC density (CD31 or EMCN+) and abnormal or enlarged sinusoids around central veins (delimited by yellow dashed lines). **m**, Immunostaining for CD45 shows strong accumulation of blood cells in the enlarged sinusoids connecting central veins. **n**, Violin plots showing expression of all canonical Notch pathway genes and downstream targets *(Hes1, Hey1, Hey2)* in the indicated EC clusters. **o**, Dot plot showing the frequency and average expression of all canonical Notch pathway genes and its downstream targets. **p**, Heatmap of arterial markers expression in the indicated datasets. Data are presented as mean values +/- SD. For statistics see Source Data File 1. Scale bars, 100 μm, except in j and k, 500 μm.

**Fig.2:**
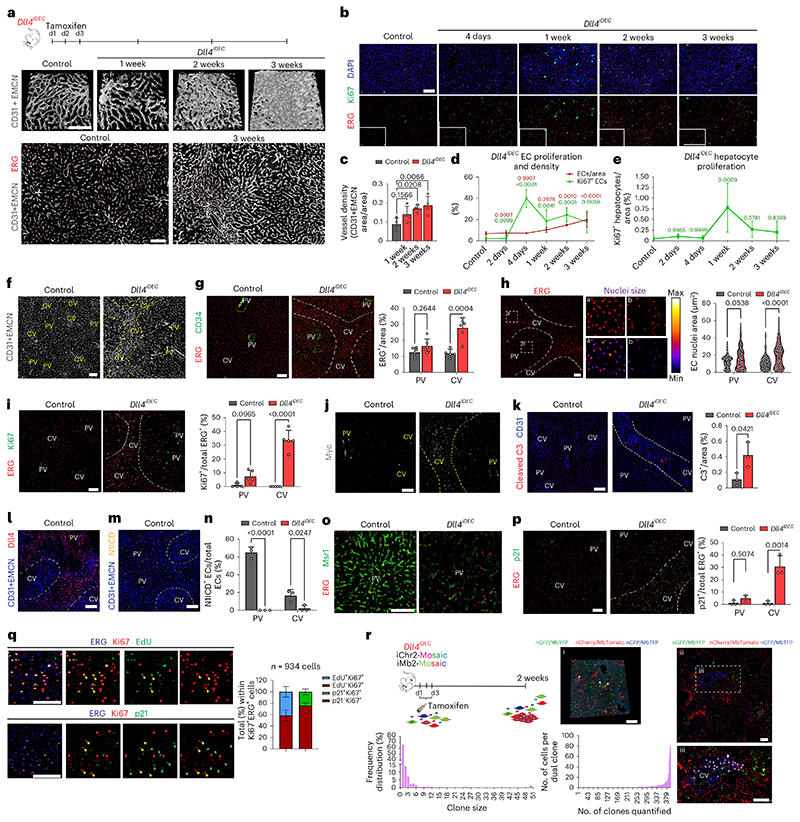
Targeting Dll4 induces heterozonal responses in liver vessels. **a**, Experimental layout for the inducible deletion of *Dll4* in Cdh5^+^ ECs *(Dll4^iDEC^)* with *Cdh5(PAC)-CreERT2.* 3D projection of confocal images from thick vibratome sections. **b**-**e**, Analysis of EC (ERG^+^ cells) and hepatocyte (ERG-DAPI^+^) proliferation (Ki67^+^) and cell number. **f**, Representative confocal micrographs showing that the abnormal vascular pattern observed in *Dll4^iDEC^* livers is located in the central vein (CV)-connecting sinusoids, but not in ECs surrounding portal veins (PV). Yellow dashed lines highlight the CV affected area. **g**, EC density in *Dll4^iDEC^* liver is higher in sinusoids connecting the CVs rather than those around PVs (CD34^+^). White dashed lines highlight the denser area. **h**, *Dll4^iDEC^* liver section showing the increase in nuclei size mainly in CV-connecting sinusoids. White dashed lines highlight the area with higher EC density and with larger EC nuclei. Higher magnification pictures of insets a and b together with pseudocoloring of nuclear sizes (lower panels) show differences in nuclei size between CV and PV areas, respectively. Violin plots reflecting changes in cell nuclei sizes. **i**, Increased EC proliferation (Ki67’ERG’) in *Dll4^iDEC^* liver, particularly in the sinusoids connecting the CVs.**j**, Myc protein is upregulated mainly in ECs (ERG^+^ cells) around the CVs after *Dll4* deletion. **k**, Increased apoptosis (cleaved caspase-3 (C3)) in CV areas upon *Dll4* deletion. **l**,**m**, Dll4 and activated N1ICD (V1744) protein are mostly present in arterial PV areas, while being mostly undetectable in venous CV areas. **n**, *Dll4* deletion leads to loss of N1ICD (Val1744) activation in liver ECs. **o**, Msr1 immunostaining showing loss of arterial identity in *Dll4^iDEC^* vessels. **p**, p21 expression in *Dll4^iDEC^* liver ECs (ERG^+^) is also higher in the sinusoids around the CVs. **q**, *Dll4^iDEC^* Ki67’ liver ECs are actively dividing in S phase (EdU^+^Ki67^+^ERG^+^, yellow arrowheads in upper panel), and a small fraction of proliferating ECs (Ki67^+^ERG^+^) also expresses p21 protein (p21^+^Ki67^+^ERG^+^, yellow arrowheads in lower panel). **r**, Dual ifgMosaic single-cell clonal tracking after *Dll4* deletion. Images showing representative dual-labeled EC clones (yellow and white arrowheads in i and asterisks in iii). Data are presented as mean values ± s.d. For statistics, see Source Data File 1. Scale bars, 100 μm.

The previous RNA-seq and histological data revealed the adult liver endothelium as the most reactive vascular bed to the targeting of Dll4-Notch signaling. Rats and chimpanzees treated with anti-Dll4 antibodies also developed significant liver vascular neoplasms and disease^5,8^; therefore, we focused our analysis on this organ. To gain deeper insight, we performed a high-resolution spatiotemporal phenotypic and transcriptomic analysis after targeting Dll4 for 2 days to 3 weeks. In contrast to targeting Dll4 during angiogenesis, targeting Dll4 in liver sinusoidal ECs (LSECs) for 48 h, which abolishes the generation of cleaved N1ICD, did not induce major transcriptomic changes (only 11 differentially expressed genes) or vascular phenotypic changes (Extended Data Fig. 2a-e). Gene set enrichment analysis (GSEA) revealed upregulation of only a few E2F and Myc target genes at this time point (Extended Data Fig. 2f-h). The increase in vascular density after targeting Dll4 was relatively slow and progressive, only becoming noticeable 1 week after genetic deletion (Fig. 2a-c). Endothelial proliferation peaked at day 4 and was sustained after, leading to a progressive increase in vascular density and the total number of ECs (Fig. 2b-d). Proliferation of neighboring hepatocytes was also increased, peaking after the peak in endothelial proliferation (Fig. 2e), suggesting that *Dll4^KO^* ECs secrete angiocrine factors inducing hepatocyte proliferation, as shown previously during liver regeneration^10^.

The effect of Dll4 targeting was, however, notably heterogeneous and zonal. Only vessels around the central veins and with a known venous identity^11^ had a higher number of ECs (Fig. 2f,g), larger nuclei (Fig. 2h), and expression of cell-cycle (Fig. 2i,j) and apoptosis (Fig. 2k) markers. Therefore, the previously reported anti-Dll4-driven liver histopathology and increase in cell proliferation^8^ is now found to be mainly associated to the central-vein sinusoids, which become enlarged and full of blood cells (Extended Data Fig. 2i-m). Paradoxically, the portal-vein sinusoids, which have arterial identity and the highest Dll4 expression and Notch activity (Fig. 2l-n and Extended Data Fig. 2n,o), showed a minor increase in EC proliferation (Fig. 2i) despite a significant loss in the expression of arterial genes (Fig. 2o and Extended Data Fig. 2p). Besides the cell-cycle marker Ki67, we also analyzed more specific S-phase (EdU) and cell-cycle arrest/senescence (p21) markers. This analysis revealed expression of p21 in 30% of *Dll4^iDEC^* ECs in the venous vessels around the central veins (Fig. 2p). Among Ki67^+^ ECs, 40% were positive for EdU and 25% were positive for p21 (Fig. 2q). This shows that there is a mix of productive cell division (EdU^+^) and arrest (p21^+^) after Dll4 loss in liver ECs. Pulse-chase single-cell ifgMosaic tracking revealed that relatively few of the Ki67^+^ ECs had the ability to divide and clonally expand after Dll4 targeting, with some cells dividing 6 to 50 times more than their neighbors (Fig. 2r). All of these progenitor cells were located in the sinusoids around central veins (Fig. 2r,iii).

### Loss of Notch1 or Rbpj in LSECs induces hypermitogenic arrest

**Extended Data Fig. 3:**
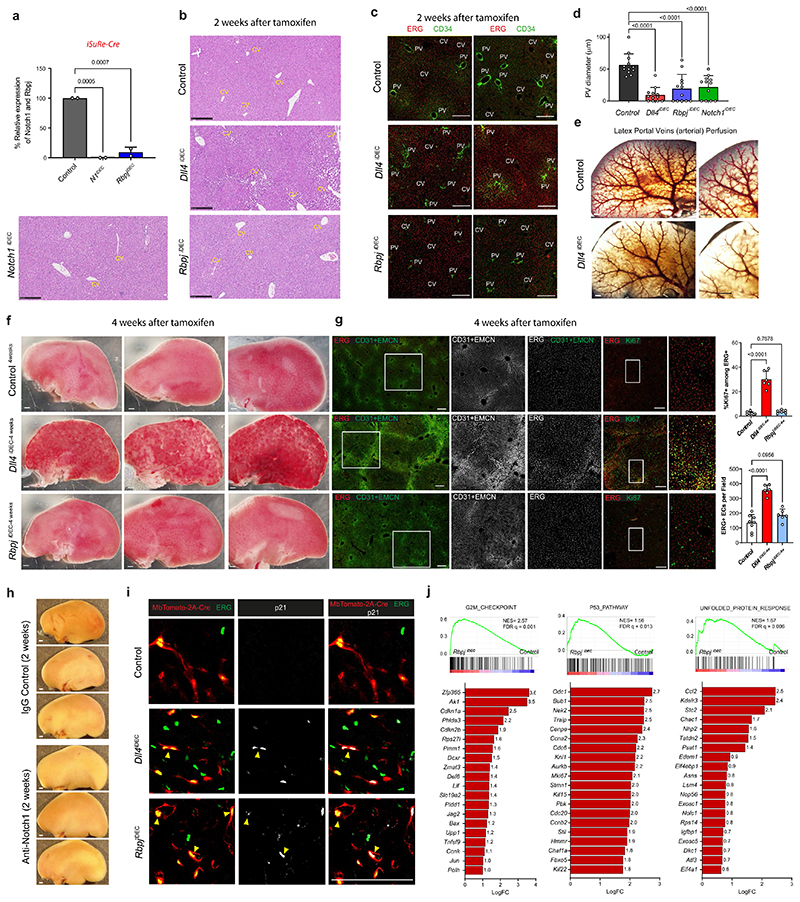
The non-pathologic and arrested endothelial status of *Rbpj* mutant ECs is linked to general replicative or cellular stress genetic pathways. **a**, *Rbpj* and *Notch1* genes were efficiently deleted in liver quiescent ECs as shown by their relative RNA-seq counts per million. Note for *Rbpj* gene only deleted exons reads were quantified. **b**, Representative Hematoxylin and Eosin staining images of liver sections showing strong liver sinusoidal dilation around central veins (CV) and pathology in *Dll4^iDEC^* but not in *Notch1^iDEC^* or *Rbpj^IDEC^* mutants. **c,d**, Confocal micrographs and associated quantifications showing a reduction in the caliber of CD34+ distal portal veins in cryosections. **e**, Latex perfusion casts of portal veins (PV, arterial) showing reduced caliber and branching complexity of the distal branches in the mutants. **f**, Stereomicroscope pictures of livers from animals with the indicated genotypes revealing that only *Dll4* deletion induces significant pathology. **g**, Confocal micrographs of cryosections showing the abnormal vasculature after *Dll4*, but not *Rbpj*, deletion in ECs. Vessels labelled with CD31 and EMCN (membrane signal, higher in CVs sinusoids) and ERG (EC nuclei). **h**, Stereomicroscope images of Anti-Notch1 treated livers show no vascular pathology. **i**, Confocal micrographs of liver sections showing that binucleated *Dll4^iDEC^* and *Rbpj^iDEC^* ECs are p21+. Yellow arrowheads indicate p21+ binucleated EC events. **j**, List of the top 20 upregulated genes in *Rbpj* mutant ECs within the indicated gene sets from the GSEA Hallmark analysis. Data are presented as mean values +/- SD. For statistics see Source Data File 1. Scale bar, 250pm in all except e, f and h, 1 mm.

**Fig.3:**
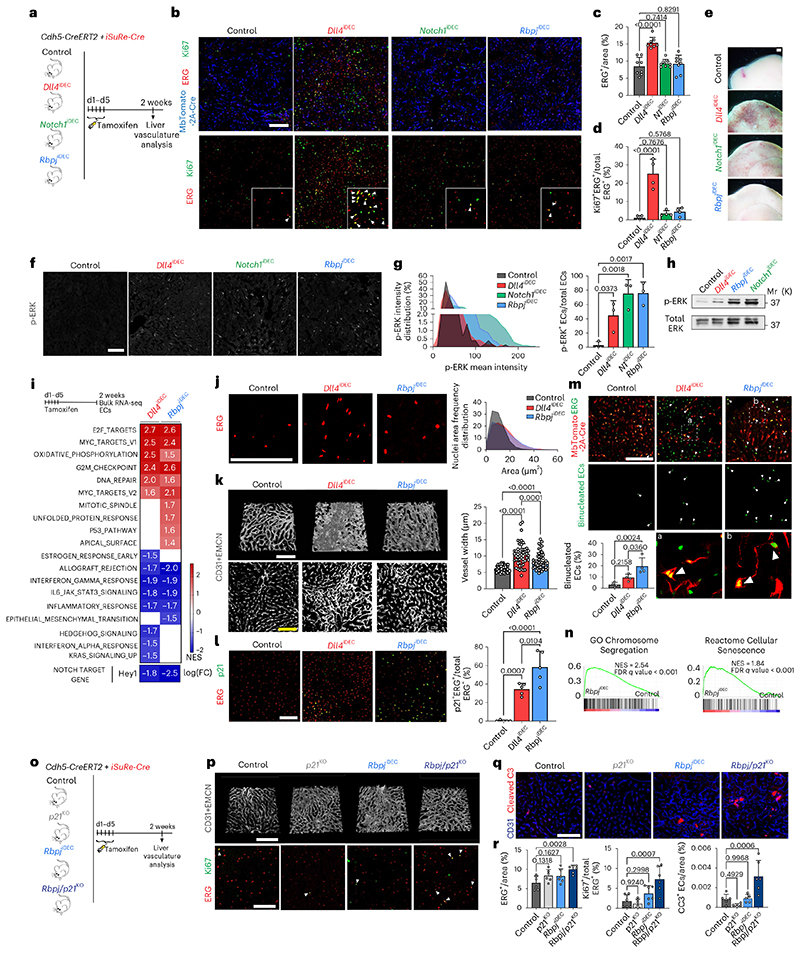
Deletion of *Rbpj* or *Notch1* in liver quiescent blood vessels does not phenocopy *Dll4* deletion. **a**, Experimental layout for the inducible deletion of *Rbpj(Rbpj^iDEC^),Notch1 (Notchi^iDEC^)* and *Dll4 (Dll4^iDEC^)* in Cdh5^+^ ECs. All mice contained the *Cdh5(PAC)-CreERT2* and *iSuRe-Cre* (expressing MbTomato-2A-Cre) alleles to ensure genetic deletion of the floxed alleles. **b**-**d**, Increased EC density (ERG^+^ per field) and proliferation (Ki67^+^ERG^+^/ERG^+^) were observed only in *Dll4^iDEC^* liver ECs. **e**, Gross liver pathology is observed exclusively in *Dll4^iDEC^* livers. **f**-**h**, p-ERK immunostaining and whole-liver western blot showing that the frequency of p-ERK-expressing ECs and intensity levels increase in the mutants, particularly the *Notch1* and *Rbpj* mutants. **i**, Heatmap with the normalized enrichment score (NES) from significant hallmark analysis (FDR *q* value < 0.05) by GSEA from bulk RNA-seq data. FC, fold change. **j**, Mutant liver ECs have a larger nuclei size than control liver ECs. **k**, Vascular (CD31^+^) dilation or expansion is more pronounced in *Dll4^iDEC^* mutants. **l**, p21 expression in ECs (p21^+^ERG^+^) is more increased in *Rbpj^iDEC^* mutants. **m**, Binucleated cells (white arrowheads) identified in *Dll4^iDEC^* and *Rbpj^iDEC^* mutants. High magnification of insets a and b are shown at the bottom. **n**, GSEA analysis shows a positive and significant enrichment in Chromosome Segregation-related and Cellular Senescence-related genes in *Rbpj^iDEC^* mutant liver ECs as shown by the NES. **o**, Experimental layout for the inducible deletion of *Rbpj* in a *p2l^KO^* background. **p**, 3D projection of thick vibratome sections showing the endothelial surface marker CD31 and EMCN, and proliferation (Ki67) analysis in ECs (ERG^+^). **q**, Analysis of the apoptosis marker cleaved caspase-3. **r**, The absence of p21 in a *Rbpj^iDEC^* background results in a modest increase in EC density (ERG^+^), but both EC proliferation (Ki67’ERG’) and apoptosis (cleaved caspase-3, CC3) are significantly increased. Data are presented as mean values ± s.d. For statistics, see Source Data File 1. Scale bars, 100 μm, except **e**, 1 mm.

Notch ligands and receptors can be targeted with a range of pharmacological compounds and antibodies^4–7^, and so far only Dll4-targeting antibodies have been reported to cause major vascular disease^5,8^. In contrast, genetic deletion of *Notch1* or *Rbpj* in mice has been suggested to cause vascular phenotypes very similar to the genetic deletion of *Dll4*, during angiogenesis and in adult vessels ^12–15^. Therefore, we investigated if deleting *Notch1* or *Rbpj*, the master regulator of all Notch receptor signaling, induced vascular pathology similar to that induced by the loss of Dll4 (Fig. 3a). Surprisingly, *Notch1* and *Rbpj* deletion for 2 weeks or 4 weeks did not significantly increase EC proliferation and related vascular pathophysiology (Fig. 3b-e and Extended Data Fig. 3a-g), despite these mutant cells having even higher activity of phosphorylated extracellular signal-related kinase (p-ERK) than ECs lacking Dll4 (Fig. 3f-h). Livers treated with anti-Notch1 blocking antibody4 also lacked the major hallmarks of pathology observed in anti-Dll4-treated livers (Extended Data Fig. 3h). Next, we compared the transcriptome of *Dll4^iDEC^* and *Rbpj^iDEC^* vessels. ECs from both mutant lines showed a similar upregulation of genes related to cell-cycle activation and metabolism (Fig. 3i) and had enlarged nuclei (Fig. 3j). However, compared with *Dll4^iDEC^* livers, *Rbpj^iDEC^* livers had significantly less vascular expansion and organ abnormalities (Fig. 3k and Extended Data Fig. 3c-g) and stronger upregulation of p21 (Fig. 3l), a cell-cycle inhibitor frequently upregulated in senescent or hypermitogenically arrested cells^16^. We also identified a significant increase in the number of binucleated p21^+^ ECs, suggestive of replicative stress and G2 arrest of the mutant cells (Fig. 3m and Extended Data Fig. 3i). RNA-seq analysis revealed signatures of genetic pathways linked to G2/M checkpoints, chromosome segregation, and general replicative stress and senescence in *Rbpj^iDEC^* ECs (Fig. 3n and Extended Data Fig. 3j). To determine the functional effect of p21 upregulation, we analyzed compound *Rbpj^iDEC^ p21^KO^* mice (Fig. 3o). p21 loss did not affect the minor vascular sinusoid dilation seen in *Rbpj^iDEC^* livers, but did increase the frequency of cycling (Ki67^+^) and apoptotic (cleaved caspase-3^+^) cells (Fig. 3p-r), in line with the role of p21 as a cell-cycle and apoptosis inhibitor^17^, particularly in hypermitogenically activated *Rbpj^KO^* cells. This dual and paradoxical effect of p21 loss on both cell proliferation and apoptosis may explain the relatively mild increase in EC numbers in *Rbpj^iDEC^ p21^KO^* livers compared with the fully arrested *Rbpj^iDEC^* liver vessels. These results suggest that loss of Dll4 induces a reduction in Notch signaling that results in a mixed population of proliferative and arrested ECs, whereas the complete loss of Notch signaling induces mostly hyper-mitogenic arrest, without productive cell division.

### Targeting Dll4 and Notch induces incongruent cell states

**Extended Data Fig. 4:**
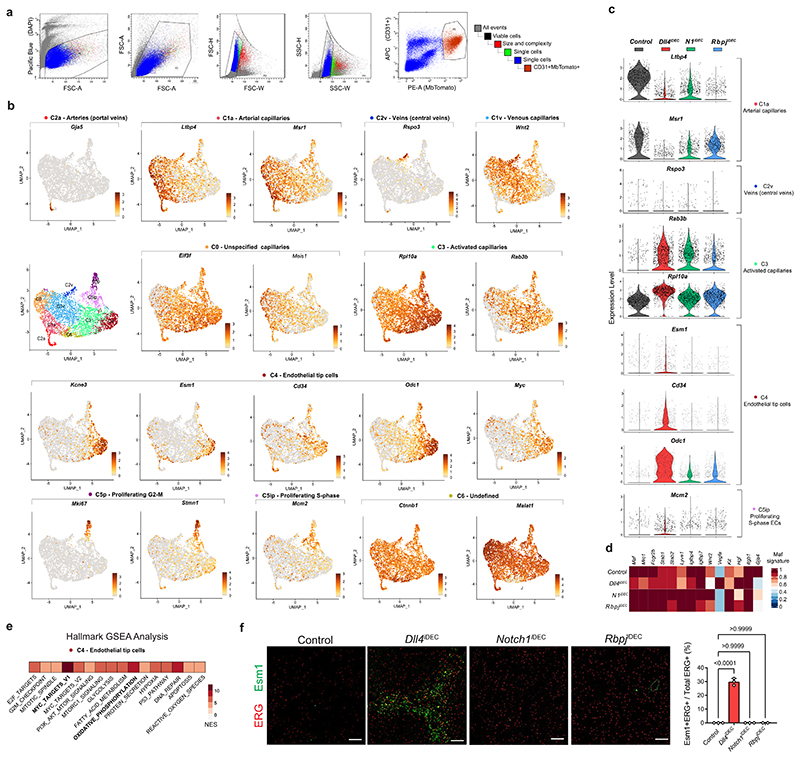
Single Cell RNA-seq analysis of Dll4 and Notch mutants. **a**, FACS plots to show the EC gating strategy. The detectors, dyes and fluorophores are indicated in the X and Y-axes. **b**, Feature plots of cluster specific or cluster enriched genes. Some clusters are also characterized by the lack of expression of a given gene. **c**, Violin plots of different cluster markers expression in indicated mutants. **d**, Maf transcription factor gene signature is downregulated in *Dll4^iDEC^* mutants. **e**, Hallmark GSEA analysis of C4 cluster showing that Myc targets and Oxidative Phosphorylation related genes are the most upregulated pathways. NES. Normalized Enrichment Score. **f**, Confocal micrographs of liver sections showing presence of Esm1+ ECs exclusively in *Dll4^iDEC^* mutants. Data are presented as mean values +/- SD. For statistics see Source Data File 1. Scale bar 100 μm.

**Fig.4:**
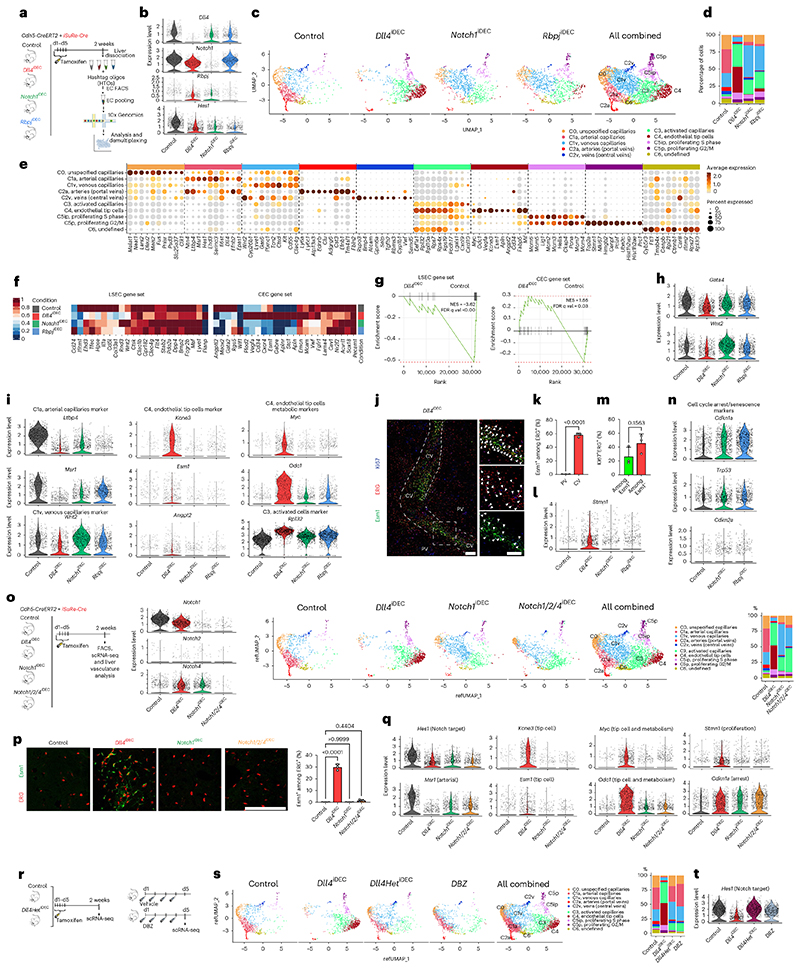
scRNA-seq analysis reveals significant differences between targeting Dll4 and Notch signaling. **a**, Experimental layout for the inducible deletion of the indicated genes in Cdh5-CreERT2^+^ ECs and collection of the iSuRe-Cre^+^ (Tomato-2A-Cre^+^) cells to ensure genetic deletion. **b**, Violin plots showing *Dll4, Notch1* and *Rbpj* mRNA expression in single cells and the subsequent downregulation of the Notch target gene *Hes1* in all mutants. **c**,**d**, UMAPs showing the ten identified clusters, and barplot showing the percentage of cells in each cluster in all samples. **e**, Dot plot showing the frequency (size) and intensity (color) of expression for the top cluster marker genes. **f**, Heatmap showing the indicated LSEC and continuous/capillary endothelial cell (CEC) gene expression signatures. **g**, Enrichment score analysis of LSEC and CEC signatures in *Dll4^iDEC^* ECs. **h**, Violin plots showing decreased *Gata4* and *Wnt2* expression only in *Dll4^iDEC^* mutants. **i**, Violin plots for some cluster marker genes. **j**,**k**, In *Dll4^iDEC^* mutants, tip cells (Esm1^+^ERG^+^) are localized in the sinusoids around CVs, but not in PV sinusoids. **l**, The global cell-cycle marker *Stmn1* is highly upregulated exclusively in *Dll4^iDEC^* liver ECs. **m**, Most of the Esm1^+^ ECs are not Ki6Z, but have Esm1-Ki67* ECs as neighbors in the CV sinusoids. **n**, Violin plots for the indicated genes and conditions. **o**, Experimental layout for the inducible deletion of the indicated genes, their violin plots, UMAPs and barplots. **p**, Expression of the tip-cell marker Esm1 in ERG^+^ ECs located in CV sinusoids. **q**, Violin plots showing that deletion of *Notch1/2/4* results in less Notch signaling *(Hes1)* and less arterial marker expression *(Msrl)*, but no induction of the tip-cell program *(Kcne3/Esm1/Myc/ Odcl)* or the proliferation marker *Stmn1.* The cell-cycle arrest marker *(Cdkn1A)* is increased. **r**, Experimental layout for the inducible heterozygous deletion of *Dll4 (Dll4Het^iDEC^)* for 2weeks or DBZ treatment for 4days in Cdh5^+^ ECs used for scRNA-seq. **s**, UMAPs and barplots obtained. **t**, Violin plots showing expression of the canonical Notch signaling target *Hes1.* Data are presented as mean values ± s.d. For statistics, see Source Data File 1. Scale bars, 100 μm.

Next, we performed scRNA-seq to identify possible differences in vascular single-cell states induced by targeting Dll4, Notch1 or Rbpj. This analysis was performed on cells expressing the *Cdh5-CreERT2* and *iSuRe-Cre* alleles18 to guarantee endothelium-specific recombination, labeling and full genetic deletion of all of the floxed genes used in this study (Fig. 4a,b and Extended Data Fig. 4a). To reduce batch effects, Tomato^+^CD31^+^ ECs were isolated on the same day from multiple control and mutant animals, tagged with different oligonu-cleotide-conjugated antibodies, and loaded in the same chip. The few mutant cells with mRNA expression of *Dll4* and *Notch1* were likely contaminants. For *Rbpj*, only exons 6-7 are deleted, leading to a less stable, but still detectable, 3’ mRNA. Altogether, the scRNA-seq data analysis showed the existence of ten clearly defined cell clusters (Fig. 4c-e and Extended Data Fig. 4b). The deletion of *Rbpj, Notch1* and *Dll4* resulted in a significant decrease in Notch signaling and *Hesl* expression (Fig. 4b) and the loss of the arterial sinusoidal capillary transcriptional C1a cluster. In agreement with this, all of these mutants had a reduction in distal portal-vein (arterial) caliber and branching complexity (Extended Data Fig. 3c-e). However, only the loss of Dll4 was able to induce a very pronounced loss of liver sinusoidal genes and capillarization^19,20^ and a tip-cell transcriptional program (C4). This program was characterized by the downregulation of *Gata4*^19^, *Maf*^21^ and the venous *Wnt2* gene expression (Fig. 4f-h and Extended Data Fig. 4d) and very high expression of the tip-cell markers *Kcne3, Esm1, Angpt2* and *Apln*, as well as *Myc* and its canonical target *Odcl* (Fig. 4i and Extended Data Fig. 4b-d). Most of the upregulated genes in the tip-cell cluster were associated with Myc metabolism, increased ribosome biosynthesis, glycolysis, mTORC1 signaling, and fatty acid and oxidative phosphorylation (Extended Data Fig. 4e). Paradoxically, *Notch1^iDEC^* and *Rbpj^iDEC^* liver ECs, in which the decrease in Notch signaling was more pronounced *(Hes1* expression in Fig. 4b), showed a more moderate metabolic activation, and most of these mutants ECs clustered in either the venous C1v cluster or the activated C3 cluster and did not reach the extreme C4 tip-cell state (Fig. 4c,d).

Histology confirmed that indeed only the *Dll4^iDEC^* mutants had a significant population of Esm1^+^ tip cells (Extended Data Fig. 4f) and that these were mostly present in the venous sinusoidal capillaries interconnecting the liver central veins (Fig. 4j,k), where EC proliferation and density are the highest (Fig. 2f-i). The upregulation of the global cell-cycle marker *Stmn1* in *Dll4^iDEC^* livers (Fig. 4l) correlated with the sixfold higher frequency of Ki67-protein^+^ cells in these mutants compared with the Notch1 and Rbpj mutants (Fig. 3d). Most Esm1^+^ tip cells were not Ki67^+^, in accordance with their higher sprouting activity and arrested nature, but had proliferating Ki67^+^ cells as close neighbors (Fig. 4j,m). *Notch1^iDEC^* and *Rbpj^iDEC^* ECs showed significant upregulation of the replication-stress/senescence markers p21 *(cdkn1a)*, p53 *(trp53)* and p16 *(cdkn2a)* (Fig. 4n). These cells undergo hypermitogenic S/ G2/M arrest (Fig. 3m,n) without becoming Kcne3^+^/Esm1^+^ sprouting tip cells (Fig. 4c,d), which is in contrast to the current understanding of sprouting angiogenesis^16,22^.

**Extended Data Fig. 5:**
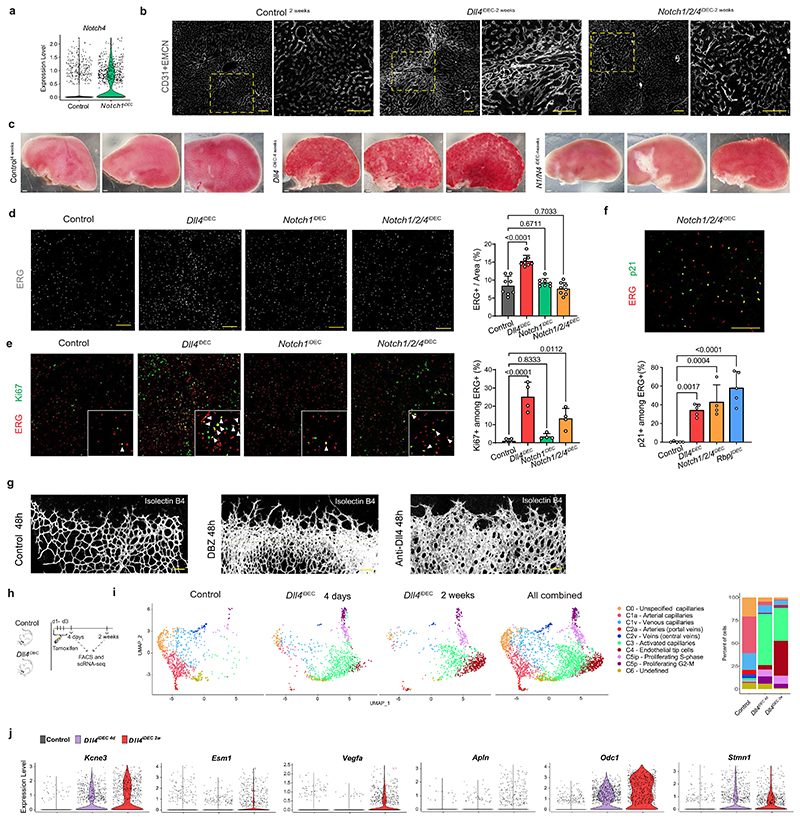
Deletion of the receptors Notch1/2/4 in liver ECs does not phenocopy *Dll4* deletion. **a**, Violin plot showing how *Notch4* expression increases after deletion of *Notch1* in liver ECs, which could compensate for its function. **b**, Confocal micrographs of liver sections showing abnormal vasculature (CD31+) around central veins (CV) in *Dll4^iDEC^* livers, but not in *Notch1/2/4^iDEC^*. Yellow dashed rectangle within left panel is to highlight the location of high-magnification images shown in right panel. **c**, Stereomicroscope images of control and mutant livers 4 weeks after tamoxifen induction of genetic deletion. **d, e**, Confocal micrographs and charts showing increased Ki67 but not productive proliferation or increased ERG+ ECs in *Notch1/2/4^iDEC^* mutants. **f**, Confocal micrograph and chart showing p21 in ERG+ ECs in *Notch1/2/4^iDEC^* mutants. **g**, When administered in postnatal day 5 pups, until day 7, DBZ has similar effects to anti-Dll4 on retina angiogenesis. **h**, Experimental layout for the *Dll4* deletion induction and scRNA-seq analysis of *Dll4^iDEC^* livers. **i**, UMAPs and barplots plot show that full loss of Dll4 signaling for 4 days leads to the loss of the arterial program (C1a) and activation and proliferation of the cells (C3 and C5), but not fully differentiated tip cells (C4).**j**, Violin plots showing that targeting Dll4 in quiescent vessels induces a fast entry in cell cycle but a relatively slow and progressive change in tip-cell related genes. Data are presented as mean values +/- SD. For statistics see Source Data File 1. Scale Bars 100 μm in all, except c, 1 mm.

*Notch1^iDEC^* livers upregulated the expression of *Notch4* (Extended Data Fig. 5a), a receptor known to partially compensate for *Notch1* deletion^23^. Deletion of *Notch1/2/4* in ECs, similarly to Rbpj loss, results in even lower *Hes1* expression and higher p21 expression (arrest); however, this does not result in the induction of tip cells (Esm1^+^/Kcne3^+^) or proliferating Stmn1^+^ cells (Fig. 4o-q and Extended Data Fig. 5b-f).

We also tested a general γ-secretase inhibitor, DBZ, which is known to block Notch signaling and elicit strong effects on tumor and retina angiogenesis^6^, similarly to anti-Dll4 treatment (Extended Data Fig. 5g). However, this compound had a very weak effect on quiescent vessels, similar to the changes seen in Dll4 heterozygous livers (Fig. 4r-t). We also observed by scRNA-seq that ECs with full loss of Dll4 signaling for only 4 days had already lost the arterial capillary program (C1a cluster) and become activated (C3 cluster), but had not yet had time to fully differentiate to tip cells (C4 cluster in Extended Data Fig. 5h-j). This suggests that in order to fully activate quiescent ECs and induce significant numbers of tip cells and vascular abnor-malization, pronounced and continuous loss of Dll4 signaling must be sustained for about 1 week, which can be achieved with genetic deletion or blocking antibodies5 but not with small-molecule inhibitors targeting Notch.

**Extended Data Fig. 6:**
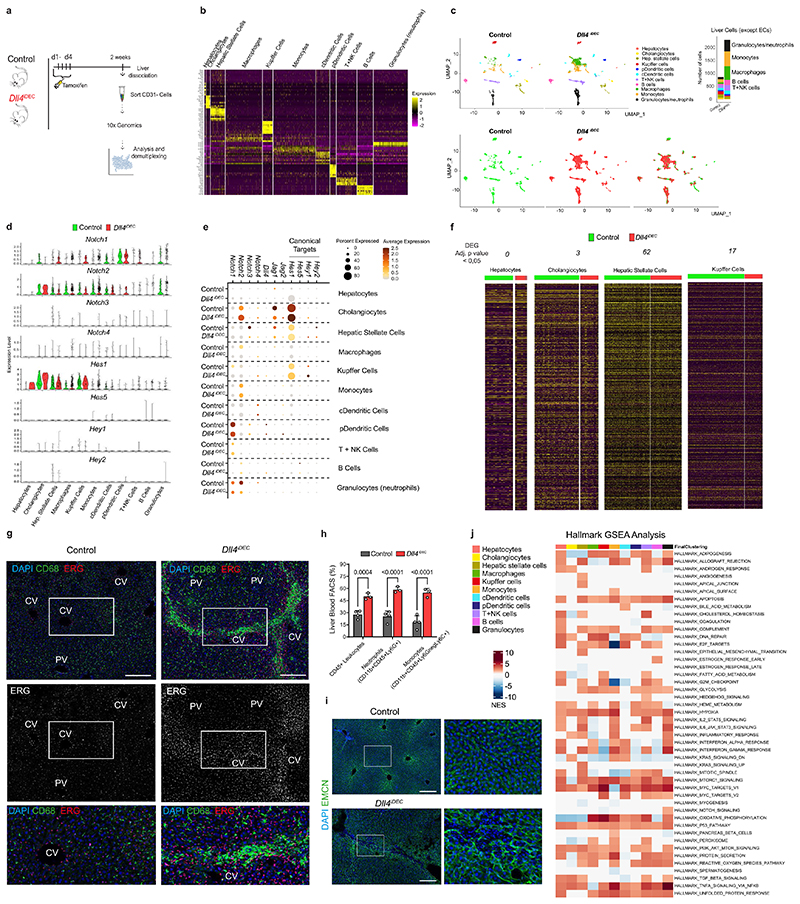
scRNA-seq analysis of other liver cell types reveals increased immune cells in *Dll4^!DIE^* livers. **a**, Experimental layout for the inducible deletion of *Dll4* in Cdh5-CreERT2+ ECs (for 2 weeks) and collection of CD31 negative cells (most hepatocytes were lost during centrifugation). **b**, Heatmap showing cluster specific gene expression and cell type identification. **c**, UMAPs showing the different cell types identified by scRNA-seq from Control and *Dll4^iDEC^* livers. Barplot showing the absolute number of each cell type in the different samples. **d, e**, Violin and dot plot showing the expression of Notch ligands, receptors and their canonical target genes in the different cell types from control and *Dll4^iDEC^* livers. **f**, Heatmap for the identified genes in the analyzed single cells revealing few differentially expressed genes in the limited number of cells analyzed. **g**, Confocal images of liver sections showing increased number of CD68+ cells in *Dll4^iDEC^* livers, particularly in the enlarged and proliferative central veins sinusoids. **h**, Quantification of different liver blood cell types by FACS. **i**, Blood (DAPI+ EMCN-) accumulation throughout the enlarged and abnormal central veins sinusoids (EMCN+). **j**, GSEA Hallmark analysis performed for every single cell type show positive or negative enrichment in different hallmarks after endothelial *Dll4* deletion for 2 weeks and subsequent organ pathology. Data are presented as mean values +/- SD. For statistics see Source Data File 1. Scale bar, 200 μm.

**Extended Data Fig. 7:**
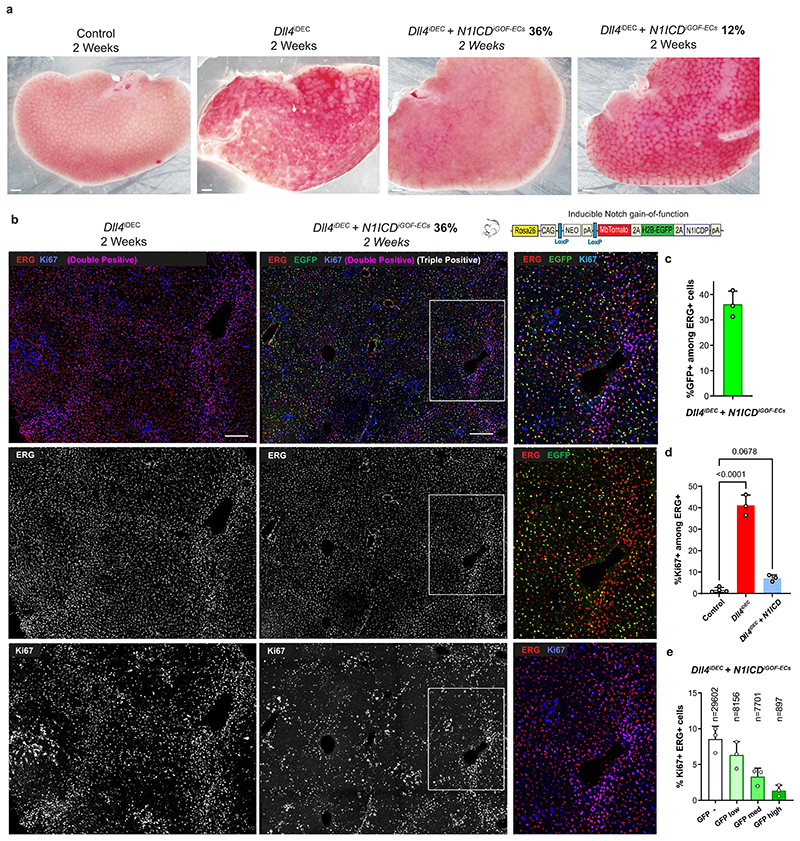
Expression ofN1ICD in *Dll4^UDCC^* mutant ECs prevents their proliferation and organ pathology. **a**, Stereomicroscope pictures showing the different degrees of liver pathology when *Dll4* is deleted, or after reconstituting transcriptional Notch activity driven by expression of the Notch intracellular domain in 12% or 36% of the liver ECs. This transgenic allele is much more difficult to recombine than the *Dll4* allele and is mosaicly expressed. **b**, Confocal micrographs showing decreased endothelial proliferation (ERG+/ Ki67+ in Dll4 mutant cells after expressing the Notch1 intracellular domain (N1ICD, nuclei EGFP+ ERG+ **c-e**, Charts showing the quantitative analysis of images like shown in b. Note that Ki67 labelling frequency decreases in animals expressing the *N1ICD* allele (EGFP+ cells), particularly in the cells with highest expression of EGFP (highest expression of N1ICD). Data are presented as mean values +/- SD. For statistics see Source Data File 1. Scale bars, 1 mm in a and 200 μm in b.

The difference between the liver vascular phenotypes of Dll4 and Notch receptor mutants could be also due to a role of the ligand, and not the receptors, on signaling to adjacent liver cells. scRNA-seq analysis of all other liver cell types revealed that hepatocytes did not express significant amounts of Notch receptors (Extended Data Fig. 6a-e). Hepatic stellate cells, Kupffer cells (stellate macrophages) and some other blood cell types expressed Notch receptors, but their target genes were not significantly downregulated by endothelial *Dll4* deletion, suggesting that this ligand mainly signals within ECs (Extended Data Fig. 6d-f). Single-cell data analysis revealed a significant increase in leukocytes in *Dll4^iDEC^* livers, particularly monocytes, neutrophils and macrophages (Extended Data Fig. 6c), presumably due to the vascular pathology and the subsequent abnormal blood flow that leads to the accumulation of these cells and an organ pathology signature (Extended Data Figs. 2j,m and 6c,g-j). Remarkably, EC-specific expression of N1ICD rescues the major hallmarks of the *Dll4^iDEC^* vascular pathology at the organ and single-cell levels (Extended Data Fig. 7). These data suggest that it is not the loss of Dll4 signaling to non-ECs that causes the difference between *Dll4^iDEC^* and *Notch1/2/4^iDEC^ or Rbpj^iDEC^* mutants. It also confirms that it is the partial downregulation of the Dll4-Notch transcriptional program in ECs, which is not matched by the complete loss of Notch receptors or Rbpj, that causes the liver vasculature abnormalization and subsequent pathology.

### Deletion of all other Notch ligands does not elicit pathology

**Fig.5:**
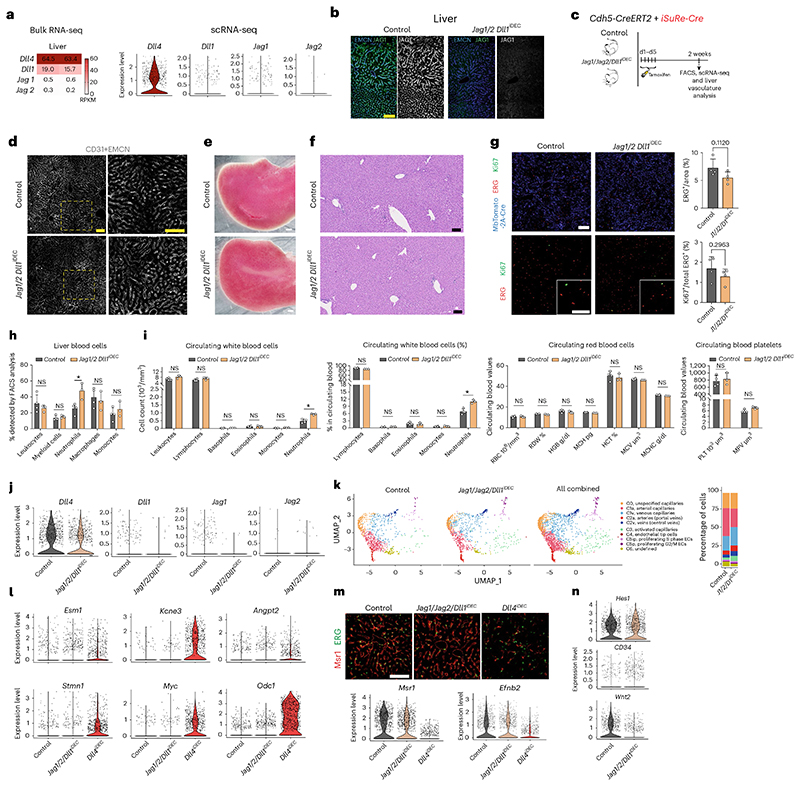
Deletion of*lag1,Jag2* and *Dll1* in liver ECs does not cause pathology. **a**, Heatmap of bulk RNA-seq reads and violin plot of single-cell data showing expression of all Notch ligands in liver ECs. **b**, Despite its low mRNA expression, Jag1 protein is clearly detected in the adult liver quiescent endothelium (EMCN^+^) and absent in *Jagl/Jag2/Dll1^iDEC^* mutants. **c**, Experimental layout for the inducible deletion of*lagl,Jag2* and *Dll1* in Cdh5^+^ ECs. **d**, CD31 and EMCN^+^ immunostaining shows no vascular architecture changes. **e**,**f**, Macroscopic pictures and hematoxylin and eosin (H&E) staining show absence of liver pathology. **g**, Deletion of the three ligands does not lead to an increase in endothelial proliferation (Ki67^+^/ERG^+^ ECs) nor an increase in EC number (ERG^+^cells per field). **h**, Analysis by FACS of the percentage of different blood cells in livers. NS, not significant. **i**, Hematological analysis of circulating (systemic) blood cells.**j**, Violin plots showing expression of the four ligands in scRNA-seq data. **k**, UMAPs and barplot showing the ten identified clusters and the percentage of cells in each cluster in the two samples. *\Jag1/Jag2/Dll1* mutant ECs do not upregulate the tip-cell *(Esm1/Kcne3/Angpt2)*, nor metabolic *(Myc/Odcl)*, nor proliferation *(Stmn1)* transcriptional program observed in *Dll4* mutants. **m**, Immunostaining and scRNA-seq data showing that*Jag1/Jag2/Dll1* mutant ECs do not downregulate the expression of the arterial markers *Msr1* and *Efnb2.* **n**, Violin plot showing an increase in the Notch target gene *Hes1* and the arterial gene *CD34*, together with a decrease in the expression of the venous *Wnt2* gene in*Jag1/Jag2/Dll1* mutant ECs. Data are presented as mean values ± s.d. For statistics, see Source Data File 1. Scale bars, 100 μm, except **e**, 1 mm.

Besides Dll4, other Notch ligands are also expressed in liver ECs (Fig. 5a). The Notch signaling target *Hes1* is more expressed in *Dll4^iDEC^* than in *Notch1^iDEC^, Rbpj^iDEC^* or *Notch1/2/4^iDEC^* mutants (Fig. 4b,q), suggesting that the other weakly expressed Notch ligands (Jagged1, Jagged2 and Dll1) may partially compensate the loss of Dll4 and induce residual Notch signaling essential for the induction of the tip-cell state. Notably,*Jagged1* mRNA was barely detectable in bulk or scRNA-seq data of quiescent liver ECs (Figs. 1d and 5a), but its protein was clearly expressed in liver vessels (Fig. 5b). Deletion of all three ligands *(Jagl, Jag2* and *Dll1)* did not alter vascular morphology, induce pathology, or increase the frequency of Ki67^+^ cells, confirming that Dll4 is the main Notch ligand in quiescent vessels (Fig. 5c-g). Liver blood profiling revealed an increase in the percentage of neutrophils, but this was also seen in circulating blood, suggesting a systemic rather than organ-specific role of these ligands (Fig. 5h,i). In agreement with this, scRNA-seq data analysis confirmed that most mutant ECs remained quiescent and did not become activated or form tip cells (Fig. 5j-l). Moreover, deletion of *Jag1,Jag2* and *Dll1* in ECs did not compromise the portal sinusoid arterial identity (Fig. 5k,m), instead revealing a slight increase in the Notch signaling target *Hes1* and the arterial gene *CD34*, together with a very pronounced decrease in the expression of the venous-enriched *Wnt2* gene (Fig. 5n). This counterintuitive increase in Notch signaling was also observed previously after the loss of Jagged1 during angiogenesis^24^.

### Myc loss prevents *Dll4^iDEC^* transcriptional states but not pathology

**Fig.6:**
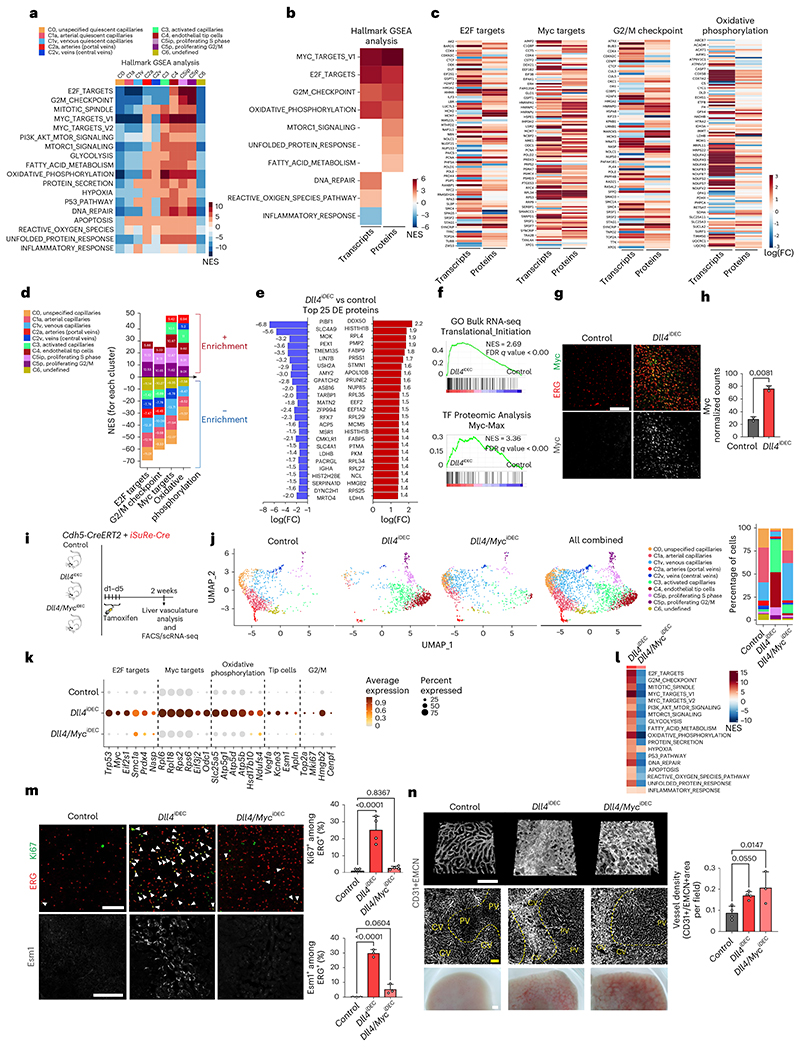
Myc loss prevents the *Dll4^KO^* endothelial activation and single-cell states but not vascular pathology. **a**, GSEA hallmark analysis for each single-cell cluster. b, GSEA hallmark analysis performed with the *Dll4^iDEC^* bulk proteome and transcriptome. c, Heatmaps showing log(fold change) ofgenes and proteins belonging to different sets. d, Barplot showing the NES in each single-cell cluster for the indicated gene sets. e, Barplot with the top differentially expressed (DE) proteins in *Dll4^iDEC^* livers. f, Enrichment analysis showing a significant positive enrichment in translational initiation-related genes and proteins encoded by genes that are regulated by the Myc-Max transcription factors. g, Micrographs showing immunostainings for the Myc protein, which is upregulated in liver ECs (ERG^+^ cells) after *Dll4* deletion. h, *Myc* mRNA expression (normalized counts from bulk RNA-seq). i, Experimental layout for the inducible deletion of *Dll4* and *Myc* in Cdh5^+^ and iSuRe-Cre^+^ ECs and scRNA-seq analysis. j, UMAPs and barplot showing the ten identified clusters and the percentage of cells for each cluster in the different samples. k, Dot plot of the top upregulated genes in *Dll4^iDEC^* liver ECs belonging to the indicated gene marker groups. l, GSEA hallmark analysis showing the decreased expression of most gene sets in *Dll4/Myd^iDEC^*. m, Double deletion of *Dll4* and *Myc* in ECs results in a significant reversion of proliferation (Ki67^+^ERG^+^ cells) and Esm1^+^ expression (Esm1^+^ERG^+^) to control levels. n, 3D confocal micrographs from thick vibratome sections (top) or thin sections (bottom), and liver macroscopic pictures showing vessel enlargement and liver pathology in *Dll4/Myd^iDEC^* mutants similarly to *Dll4^iDEC^* mutants. Data are presented as mean values ± s.d. For statistics, see Source Data File 1. Scale bar, 100 μm, except n lower panel, 1 mm.

Next, we aimed to determine the molecular mechanisms responsible for the unique EC activation, tip-cell signature, and vascular pathology induced by targeting Dll4. As mentioned above, *Myc* and its target *Odc1* were among the most strongly upregulated genes in Dll4 mutant ECs, compared with Notch1 and Rbpj mutants. Myc is known to activate important ribosome biogenesis and protein translation pathways, favoring cell growth^25^. *Dll4^iDEC^* livers showed upregulation of a large range of canonical E2F, Myc, mTORC1 and ribosomal *(Rpl)* genes, particularly in the activated, proliferating and endothelial tip-cell clusters (Fig. 6a and Extended Data Fig. 4). This hypermetabolic transcriptional status was confirmed by mass spectrometry (MS) analysis of protein lysates obtained from freshly isolated liver ECs (Fig. 6b-f), providing a high-depth proteomic analysis of the endothelial tip-cell state induced by targeting Dll4. We also independently confirmed *Myc* mRNA and protein upregulation in *Dll4^KO^* vessels (Fig. 6g,h).

**Extended Data Fig. 8:**
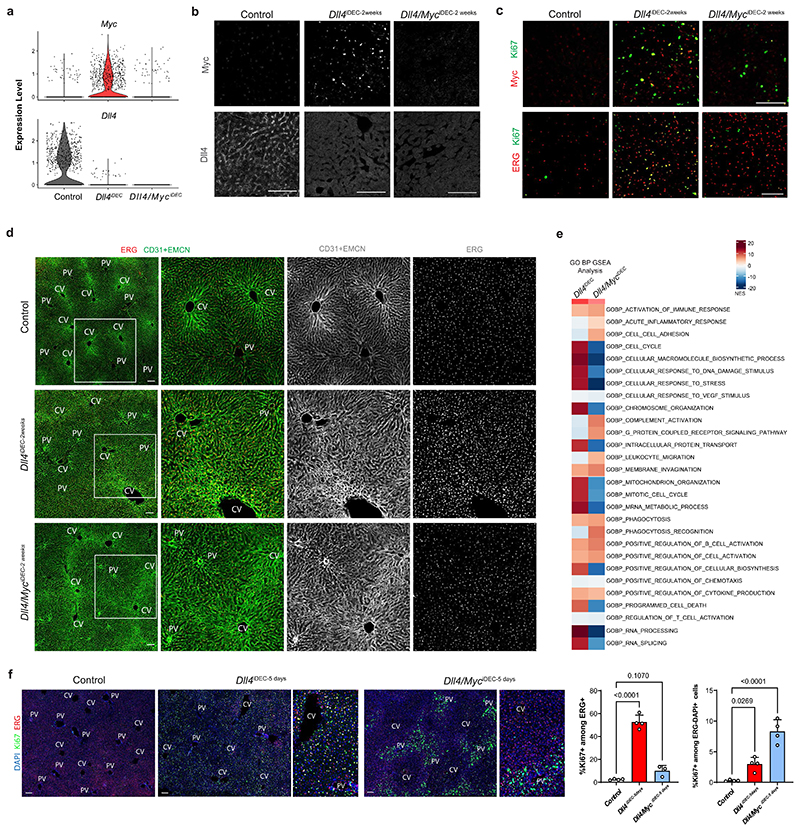
Deletion of *Myc* in *Dll4^,DCC^* mutants blocks EC proliferation but not vessel enlargement and malfunction. **a**, scRNA-seq violin plots show deletion of *Dll4* and *Myc* genes in the indicated samples. **b**, Confocal micrographs of liver sections showing Dll4 absence and Myc upregulation in *Dll4^iDEC^* livers and absence of Dll4 and Myc in the double *Dll4/ Myc^lDEC^* mutants. **c**, Confocal micrographs of liver sections showing absence of EC proliferation (Ki67+ERG+) and endothelial Myc expression in the double *Dll4/ Myc^iDEC^* mutants. **d**, Micrographs showing increased vascular (CD31+ EMCN) density and abnormalization in *Dll4/Myc^lDEC^* mutants despite similar number of ECs (ERG+) to control livers. **e**, scRNA-seq Gene Ontology (GO) analysis of *Dll4^iDEC^* and *Dll4/Myd^iDEC^* liver ECs showing that the loss of Myc strongly downregulates some of the biological processes upregulated in *Dll4^iDEC^* cells, but not processes related with inflammation. **f**, Confocal micrographs from livers showing a loss of EC proliferation (Ki67+/ERG+) but not neighbouring hepatocytes proliferation (Ki67+/ERG-/DAPI+) after combined loss of Myc and Dll4 for only 5 days. Data are presented as mean values +/- SD. For statistics see Source Data File 1. Scale bars, 100 μm.

Next, we investigated the implication of Myc in the *Dll4^iDEC^* transcriptional program and subsequent vascular-related pathology. Myc loss (in *Dll4/Myc^iDEC^* animals) almost entirely blocked the EC activation induced by *Dll4* loss, and very few ECs were in the activated (C3) and tip-cell (C4) clusters (Fig. 6i-l and Extended Data Fig. 8a). Consistent with the scRNA-seq data, frequencies of proliferating (Ki67^+^) and tip (Esm1^+^) cells in *Dll4/Myc^iDEC^* mutants were similar to those in wild-type animals (Fig. 6m and Extended Data Fig. 8b, c). Myc activity is thus essential for the strong metabolic and biosynthetic phenotype of *Dll4^KO^* liver ECs and the appearance of the abnormal cell states. Surprisingly, despite this strong transcriptional and cell-state reversion to a quiescent state, *Dll4/ Myc^iDEC^* mutant vessels were still highly abnormal and dilated (Fig. 6n and Extended Data Fig. 8d). The vascular abnormalities in *Dll4/ Myc^iDEC^* mutant livers were not in accordance with their more quiescent scRNA-seq profile (Fig. 6j-l), nor with the significantly lower frequencies of Ki67^+^ and Esm1^+^ cells (Fig. 6m). Interestingly, *Dll4/ Myc^iDEC^* livers retained hallmarks of tissue hypoxia and inflammation (Fig. 6l and Extended Data Fig. 8e) and had strong activation of surrounding hepatocytes already 5 days after deletion (Extended Data Fig. 8f), despite having a quiescent endothelium. Altogether, these data indicate that the vascular structure abnormalization observed in *Dll4* mutant livers is not driven by the detectable changes in endothelial transcriptional programs or the proliferative and tip EC states.

### Anti-VEGFA treatment prevents the *Dll4^IDEC^* pathology with less effect on transcription

**Extended Data Fig. 9:**
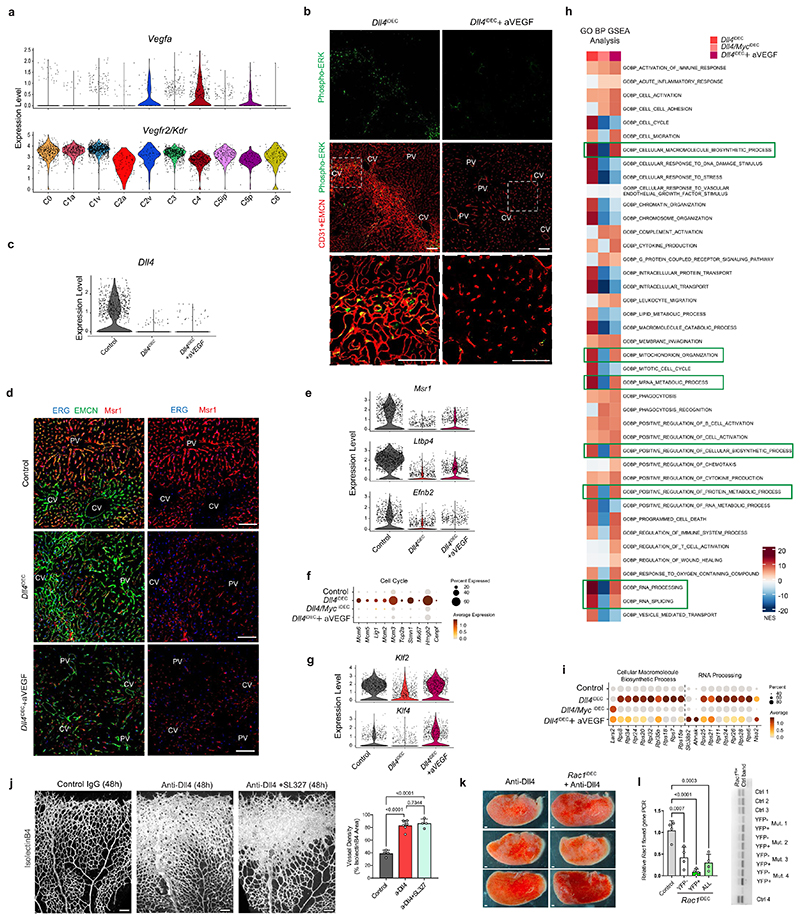
Anti-VEGFA in *Dll4^u^’^lC^* mutants reverses EC proliferation, liver pathology and vessel enlargment but not most of the *Dll4^KO^* genetic programme. **a**, scRNA-seq violin plot showing *Vegfa* upregulation in the *Dll4^iDEC^* specific endothelial tip cell cluster (C4) and *Kdr/Vegfr2* expression. **b**, Confocal micrographs of liver sections showing the absence of phosphorylation of the VEGFA target ERK after VEGFA blockade. **c**, scRNA-seq violin plot showing *Dll4* deletion in the indicated samples. **d**,Confocal micrographs of liver sections showing the loss of the capillary marker Msr1 in *Dll4^iDEC^+* anti-VEGFA samples as observed in *Dll4^iDEC^* liver ECs. **e**, Violin plots for the arterial markers *Msr1, Ltbp4* and *Efnb2* showing that anti-VEGFA does not rescue the arterial identity of cells after *Dll4* deletion. **f**, Dot plot of cell-cycle genes showing that ECs are mostly quiescent in *Dll4^iDEC^+* anti-VEGFA samples. **g**, Violin plots showing single cell expression of *Klf2* and *Klf4* genes. **h**, scRNA-seq Gene Ontology (GO) analysis showing that the loss of *Myc* more strongly downregulates the genes and biological processes uppregulated in *Dll4^iDEC^* mutants than the blockade of VEGFA. **i**, Dot plot of Cellular Macromolecule Biosynthetic process and RNA processing GO gene sets showing that they are still active in *Dll4^iDEC^+* anti-VEGFA samples. **j**, SL327 treatment for 48h in pups from postnatal day 5 to 7 does not prevent the increase in vascular density and angiogenesis observed after anti-Dll4. **k**, Stereomicroscope images of livers showing vascular and organ pathology in all conditions. **l**, Semiquantitative DNA PCR showing *Rac1* deletion efficiency in the sorted ECs (CD31+ YFP+ or CD31+YFP-) of *Rac1^iDEC^* mutants. One of the three PCR gel pictures (see Source Data File 3) is shown on the right. Data are presented as mean values +/- SD. For statistics see Source Data File 1. Scale bars, 100 μm in all except k, 1 mm.

**Fig.7:**
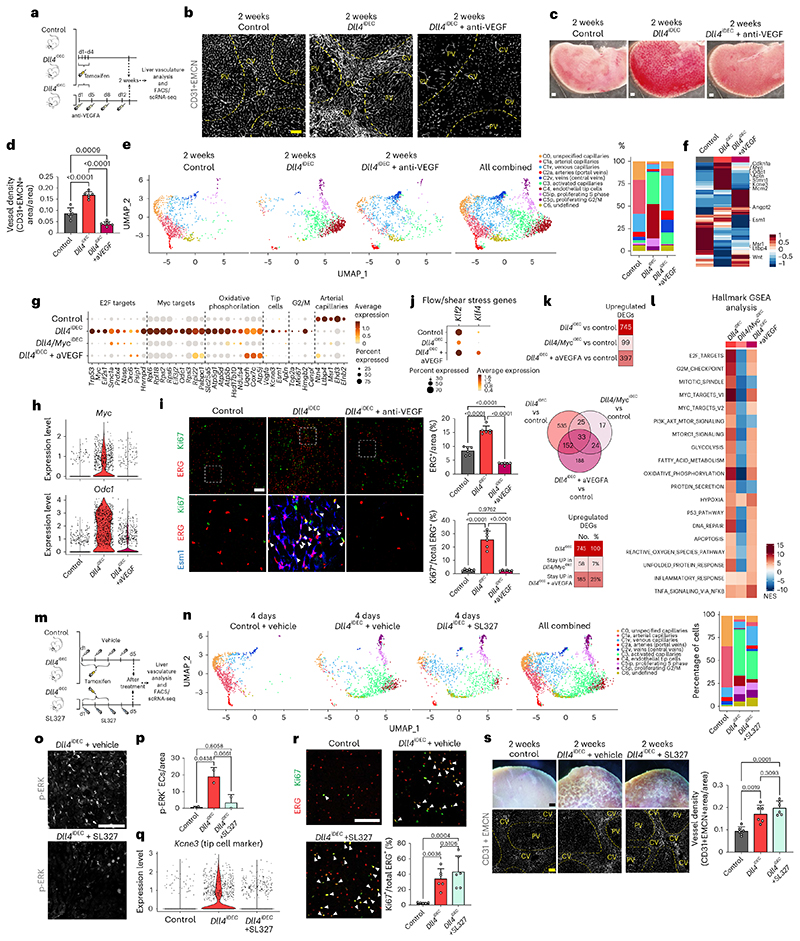
Vascular abnormalities and liver pathology are prevented by VEGFA antibody administration in *Dll4^iDEC^* mutants by ERK-independent mechanisms. **a**, Experimental layout for the inducible deletion of *Dll4* in Cdh5^+^ECs and VEGFA antibody administration. **b**, Confocal micrographs showing reduced CD31 or EMCN vascular immunostaining after anti-VEGFA treatment. **c**, Stereomicroscope liver pictures. **d**, Vessel density is reduced in *Dll4^iDEC^* mutants after anti-VEGFA treatment. **e**, UMAPs and barplot showing the identified clusters and the percentage of cells for each cluster in indicated samples. **f**, Unsupervised hierarchical clustering showing gene expression changes. **g**, Dot plot ofthe top upregulated genes for each indicated gene set. **h**, Violin plots of scRNA-seq data showing that anti-VEGFA treatment prevents the strong upregulation of *Myc* and its target *Odcl.* **i**, The total number of ERG^+^ ECs, proliferation (Ki67^+^ERG^+^) and Esm1 expression (Esm1^+^ERG^+^) return to control conditions after VEGFA antibody administration. **j**, Dot plot showing expression of flow/shear stress genes. **k**, Number of upregulated genes for each contrast and Venn diagrams showing that when compared with *Myc* loss, anti-VEGFA treatment has less effect on the *Dll4^DE^* upregulated genetic program. DEGs, differentially expressed genes. **l**, GSEA hallmark analysis confirms the more moderate effect of anti-VEGFA treatment on the *Dll4^iDEC^* genetic program when compared with *Myc* loss. **m**, Experimental layout for the inducible deletion of *Dll4* and SL327 administration. **n**, UMAPs and barplot showing the identified clusters and the percentage of cells for each cluster in indicated samples. **o,p**, The administration of an ERK/MEK signaling inhibitor (SL327) results in reduced ERK phosphorylation. **q**, Violin plot showing that SL327 treatment partially inhibits the generation of tip cells (*Kcne3*^+^). **r**, The administration of SL327 does not change the frequency of proliferating Ki67’ ECs (Ki67^+^ERG^+^). **s**, Abnormal vasculature (CD31^+^EMCN^+^) associated with liver pathology still occurs after SL327. Data are presented as mean values ± s.d. For statistics, see Source Data File 1. Scale bars, 100 μm, except in **c** and **s** upper panel, 1 mm.

Among the few GSEA hallmark pathways whose upregulation in *Dll4* mutants was not altered in *Dll4/Myc^iDEC^* vessels was the hypoxia pathway and inflammatory response (Fig. 6l and Extended Data Fig. 8e). Hypoxia is known to induce expression of vascular endothelial growth factor A (VEGFA), which can induce vascular expansion without the need for proliferation^26^. The expression of VEGFA was significantly upregulated in the *Dll4^KO^* venous tip-cell cluster (Extended Data Fig. 9a). Therefore, we explored if anti-VEGFA treatment could prevent the appearance of the activated vascular cell states, vascular enlargement and liver pathology induced by *Dll4* deletion. Unlike Myc loss, anti-VEGFA treatment reduced both the vascular expansion and the liver pathology induced by *Dll4* deletion (Fig. 7a-d and Extended Data Fig. 9b). scRNA-seq analysis confirmed the almost-complete loss of the tip-cell (C4) and proliferating (C5) single-cell states, as well as a significant reduction in the activated cell states (C3), with a general return to the quiescent cell states, with exception of the arterial state (Fig. 7e-i and Extended Data Fig. 9c-f). scRNA-seq and histology data also revealed a depletion of VEGFR2/ Kdr^+^ sinusoidal capillaries by anti-VEGFA treatment (Fig. 7b-e, i and Extended Data Fig. 9a). Anti-VEGFA treatment rescued the expression of the blood flow and shear stress responsive genes *Klf2* and *Klf4* (Fig. 7j and Extended Data Fig. 9g), suggesting a normalization of vessels and blood flow.

These results show that anti-VEGFA treatment prevents not only the appearance of the abnormal single-cell states induced by Dll4 targeting, as Myc loss also does, but also the vascular expansion and blood flow abnormalities associated with organ pathology. However, blocking VEGF had a much lesser effect than Myc loss on the *Dll4^KO^* transcriptional signature (Fig. 7k). Anti-VEGFA treatment of *Dll4^iDEC^* livers attenuated, but did not completely downregulate, many of the genes associated with metabolic and biosynthetic activities (Fig. 7l and Extended Data Fig. 9h, i). This suggests that even though *Dll4,^iDEC+^* anti-VEGFA-treated ECs are transcriptionally and metabolically more active than *Dll4/Myc^iDEC^* ECs, only the latter form abnormal and enlarged vessels that result in organ pathology.

### Inhibition of major signaling pathways did not prevent *Dll4^IDEC^* pathology

**Fig.8:**
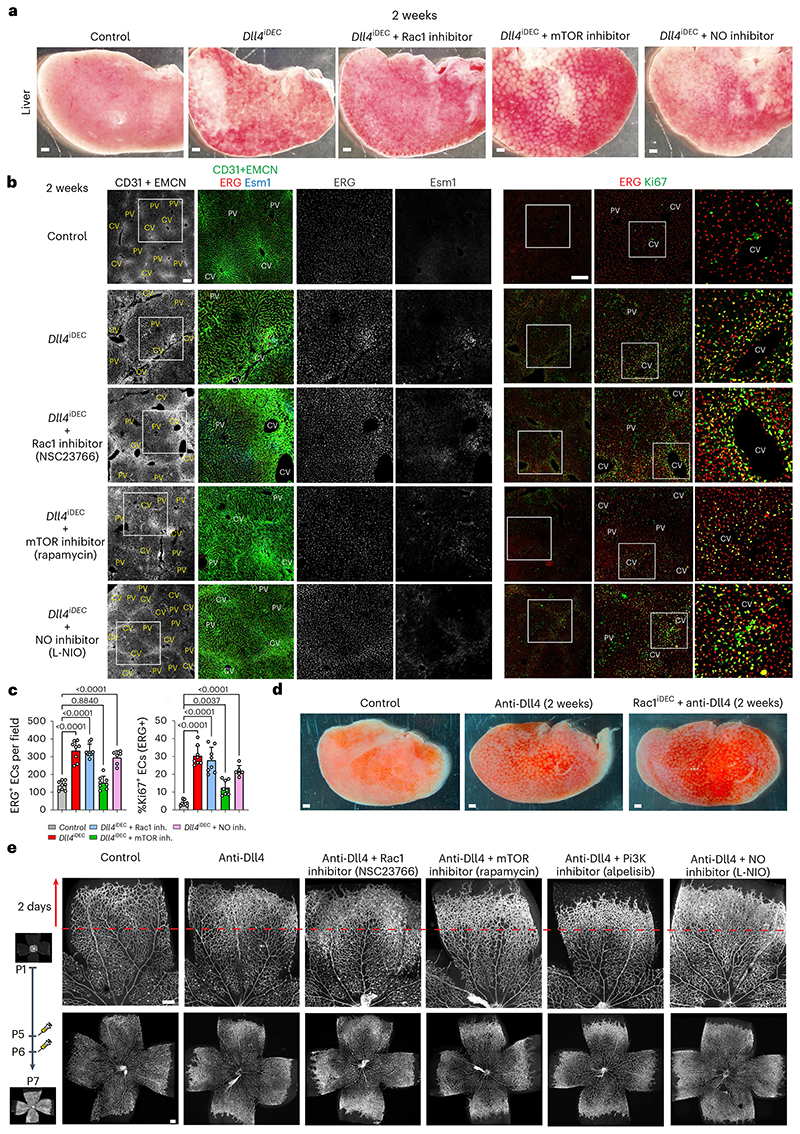
Inhibition of Rac1, mTOR and NO signaling does not prevent the vascular pathophysiology induced by Dll4 targeting. **a**, Stereomicroscope images showing adult liver vascular defects and blood accumulation after *Dll4* deletion and treatment with different inhibitors for 2weeks. **b**, Confocal micrographs showing that the expansion and abnormalization of the liver sinusoids (CD31^+^EMCN^+^), particularly around CVs, observed after *Dll4* deletion, are not prevented by the administration of the indicated compounds. On the right, images show EC (ERG^+^ nuclei) proliferation (Ki67’). **c**, Charts showing quantification of EC density/numbers and proliferation. Note that mTOR inhibitor-treated liver ECs do not proliferate significantly (same ERG^+^ content), despite a fraction being Ki67^+^. **d**, Deletion of Rac1 with Cdh5-CreERT2 in adult liver endothelium (Cdh5^+^) does not prevent the vascular pathology induced by blocking Dll4 with REGN1035. **e**, The use of the indicated inhibitors in postnatal mouse retina angiogenesis assays for 48h confirms that they do not prevent the increase in vascular expansion/density (isolectin B4 labeling) induced by anti-Dll4 antibody treatment (7.5 mg/kg). Note that 2days of angiogenesis growth correspond to the vasculature formed above the red dashed line. Data are presented as mean values ± s.d. For statistics, see Source Data File 1. Scale bars, 200 μm, except in **a** and **d**, 1 mm.

VEGFA induces many important endothelial functions that are often difficult to distinguish, such as proliferation, sprouting, cell size, survival and permeability^27–29^. VEGF is thought to execute its effects on sprouting and angiogenesis mainly through ERK signaling^30,31^. However, administration of a highly effective ERK/ MEK signaling inhibitor (SL327) had a much more modest effect than anti-VEGFA treatment, and only partially reduced the number of activated and tip ECs (Fig. 7m-s and Extended Data Fig. 9j). The VEGF-dependent vascular enlargement or expansion could be alternatively mediated by increased Rac1(ref. 32), Pi3K/mTOR (refs. 33,34) or nitric oxide (NO)^35,36^ signaling. However, the inhibition of these pathways also did not prevent the vascular pathophysiology induced by targeting Dll4 (Fig. 8 and Extended Data Fig. 9k,l). Rapamycin effectively prevented the increase in the number of ECs, but not vascular dilation and pathology. Thus, the vascular pathophysiology effects of anti-VEGFA treatment, and anti-Dll4, are broader and independent of the activity of these signaling pathways.

Overall, these results show that the genetic and pharmacological modulation of single-cell states related to endothelial dedifferentiation, activation, proliferation and sprouting often do not correlate with adult vascular phenotypes, function and ultimately organ pathology.

## Discussion

Notch is one of the most important pathways for vascular development because it enables the necessary differentiation of ECs during angiogenesis^28,3,38^. Here, we expand on previous observations that Notch also plays an important role in the homeostasis of several organ vascular beds^8,12^. Dll4 is active in all organ vascular beds, and its loss affects the transcriptome of most quiescent ECs; however, Dll4 targeting effectively activates vascular growth in only the heart, muscle and liver. Even though the existence of four Notch receptors and five ligands allows for the possibility of multiple quantitative and qualitative signaling combinations and redundancy, our results confirm that Dll4 and Notch1 are clearly the most important Notch ligand-receptor pair for maintaining the global homeostasis of ECs.

Previous work suggested that Dll4 and Notch1/Rbpj have similar functions in vascular development and homeostasis^6–8,15,23,24,39^, with only Jagged ligands shown to have opposite functions in Notch signaling and angiogenesis^24^. In this study, we show that Dll4 can have distinct functions from its receptors in vascular biology. It was possible to identify this difference only because of the use of scRNA-seq and high-resolution confocal analysis of liver vessel morphology; bulk RNA-seq analysis did not reveal significant differences between the transcriptomes of Dll4 and Rbpj mutants. The loss of Dll4, unlike the loss of Notch receptors or Rbpj, elicits a unique cascade of changes that culminates in the loss of sinusoidal marker genes and upregulation of Myc, similar to the loss of Gata4 (ref. 19). *Dll4^iDEC^* liver vessels lose all quiescent arterial and venous cell states. The arterial cells become highly activated, and the venous cells show either tip-cell or proliferating cell signatures. Paradoxically, although Dll4 loss induces a weaker loss of Notch signaling than is induced by the loss of Notch receptors or Rbpj, it elicits a much stronger metabolic activation and expansion of the liver endothelium. This may be in part related to the bell-shaped response of ECs to mitogenic stimuli, as we previously showed during retina angiogenesis^16^. Our data indicate that full loss of Notch, or Rbpj, induces stronger ERK signaling and hypermitogenic arrest associated with hallmarks of cellular senescence, whereas *Dll4^iDEC^* vessels retain a residual level of Notch signaling that instead effectively induces strong Myc-driven ribosome biogenesis and a metabolic switch toward active protein synthesis and cell growth that drives both EC proliferation and the generation of tip cells. The pro-proliferative effect of targeting Dll4 in quiescent vessels is in contrast to the hypermitogenic cell-cycle arrest that occurs after targeting Dll4 during embryonic and retina angiogenesis^16,40^, presumably a reflection of the significantly lower levels of growth factors, including VEGF, in adult organs.

Previously, a noncanonical and N1ICD transcription-independent role for Dll4/Notch in inducing Rac1 and maintaining vascular barrier function was proposed^41^. *Dll4* deletion could also affect signaling to other cell types, unlike deletion of the Notch receptors in ECs. However, our data show that the liver vascular abnormalization after targeting Dll4 can be rescued by the expression of N1ICD in ECs. This suggests that the vascular pathology is caused by the absence of Dll4 canonical signaling and transcription within the endothelium, and not due to noncanonical effects on vascular barrier function, or the loss of Notch signaling in other adjacent cell types. The observed lack of pathology in anti-Notch1-treated livers also corroborates this.

High-resolution confocal microscopy revealed the heterozonal effect of Dll4 targeting. The induction of EC proliferation and tip cells was restricted to the most hypoxic liver venous sinusoids, precisely the ones with lower expression of Dll4 and Notch. Previous research showed that liver venous sinusoids have higher baseline activity of several tyrosine kinase signaling pathways^42^, which may explain the observed zonal effect of Dll4 targeting.

The temporal analysis of the effects of Dll4 targeting on the adult liver vasculature also revealed that it takes at least 1 week for the full transcriptional reprogramming of quiescent ECs and the vascular expansion and organ pathology to become noticeable. During angiogenesis, this transcriptional and vascular morphology switch is already evident after 24 h of anti-Dll4 treatment^16^. This slow transcriptional reprogramming of quiescent ECs by Dll4 targeting may be related to the much lower levels of growth factors and nutrient availability in adult organs. The slow nature of this reprogramming may also explain the lack of effect of the small-molecule inhibitor DBZ on quiescent ECs. Unlike anti-Dll4 treatment or genetic deletion, which result in continuous loss of signaling, the less stable small-molecule inhibitor DBZ elicited no significant change in the quiescent vascular cell transcriptional states and phenotypes, whereas it is very effective during retina angiogenesis^15,16^. Anti-Notch1^4^ also did not cause liver vascular pathology, despite its strong effect on angiogenesis. These findings have implications for selecting the most effective and safest way to target Notch in clinics, including blocking antibodies that target Dll4 versus antibodies that target Notch receptors, or the use of smallmolecule inhibitors. Our data indicate that Notch receptor-targeting antibodies or small-molecule γ-secretase inhibitors do not induce significant liver vascular pathology and should be as effective as anti- Dll4 treatment at dysregulating tumor-related or ischemia-related angiogenesis, which can be beneficial in some therapeutic settings. It has also been shown that is possible to modulate the stability and pharmacokinetics of anti-Dll4 treatment to decrease its toxicity while maintaining its therapeutic and angiogenesis efficacy^5^.

Our analysis also confirms the importance of Myc for the biology of ECs in the absence of Dll4. We previously reported that *Myc* loss rescues the ability of *Rbpj^KO^* or *Dll4^KO^* ECs to form arteries^40^. Here, we show that Myc loss abrogates the generation of activated, proliferative and sprouting tip cells after Dll4 targeting, but surprisingly, this return to genetic and phenotypic quiescence is insufficient to prevent Dll4- targeting-induced vascular expansion, dysfunction and consequent organ pathology. In contrast, anti-VEGFA treatment did not completely abrogate the Dll4-targeting genetic program, but was able to prevent the associated vascular and organ pathology. However, this effect of anti-VEGFA treatment was not reproduced by inhibition of MAPK/ERK, Rac1, Pi3K/mTOR or NO signaling. This suggests a broader role for anti- VEGFA treatment in preventing pathological vascular enlargement and remodeling when combined with the anti-Dll4 antibody, that could be also related to its effect on liver EC survival. Our data suggest that the action of VEGF on vascular expansion and survival is independent of its direct effect on these signaling pathways^30,32-36^, and independent of cell proliferation and sprouting, as also previously proposed^26,43^. The sum of these findings also suggests that the recently developed bispecific antibody targeting both Dll4 and VEGF simultaneously (navicixizumab, OncXerna) may be less toxic than the use of anti-Dll4 treatment alone^44^.

**Extended Data Fig. 10:**
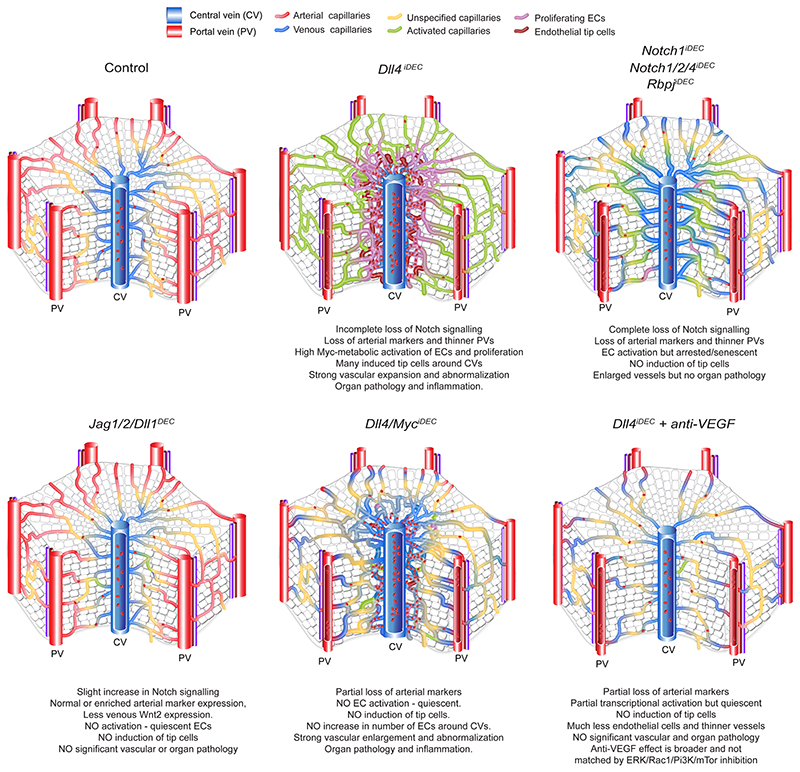
Incongruence between cell states, vascular morphology and pathophysiology. Illustration showing the main endothelial cell states and vascular phenotypes of the indicated livers. Targeting of the ligand Dll4 triggers incomplete loss of Notch signaling which results in the loss of arterial markers, reduced PV caliber and a strong Myc-driven metabolic activation associated with a well-defined cluster of proliferating and tip cells located in the sinusoids around the central veins. Dll4 mutant ECs have very high ribosome biogenesis, protein synthesis and oxidative phosphorylation favouring cell growth and metabolism. This genetic activation correlates with a significant increase in the number of proliferating and sprouting venous ECs, vascular enlargement and subsequent organ pathology associated to the abnormal blood flow in CV sinusoids. The loss of Notch receptors or Rbpj leads to complete loss of Notch signaling and also the loss of the arterial transcriptional program and reduced PV caliber, but in this case most liver sinusoidal ECs undergo an hypermitogenic cell-cycle arrest and display senescence features. In contrast to *Dll4* mutant ECs, *Notch* or *Rbpj* mutant ECs do not effectively proliferate or sprout and there is no significant vascular and organ pathology in mutant livers. Loss of all other Notch ligands leads to a mild increase in Notch signaling, without any associated vascular pathophysiology. Loss of Myc prevents most of the *Dll4* mutant transcriptional program activation and cellular states. However, even in the absence of proliferating, sprouting and activated cells, *Dll4/Myc^lDEC^* mutant livers still have abnormal and expanded CV sinusoids and significant organ pathology. Targeting VEGF only partially reduces the *Dll4* mutant genetic programs, but it is enough to prevent most of the activated and tip cell states, being ECs in a quiescent state. Anti-VEGFA also induces the very significant loss of ECs, which overall prevents the vascular enlargement and associated organ pathology. The effect of anti-VEGFA is broader and is not matched by the use of inhibitors targeting the ERK, Rac1 and Pi3k/mTor signaling.

Altogether, the data obtained with several compound mutant and pharmacological approaches show that most of the transcriptional changes and angiogenic cell states elicited by targeting Dll4 correlate with, but do not cause, vascular pathophysiology (Extended Data Fig. 10). Therefore, vascular neoplasms are not the cause of the previously reported anti-Dll4 antibody toxicity^8^. Instead, we propose that the unrelated venous sinusoid enlargement and architecture abnormalization lead to vascular malfunction, blood accumulation, inflammation and hypoxia, altogether resulting in organ pathology.

These data also raise questions about the general use of single-cell transcriptional or genetic states to describe and predict functional or dysfunctional vascular phenotypes and ultimately organ pathophysiology. A single-cell transcriptional state is only a small part of a cell’s phenotype and function.

## Methods

### Mice

The following mouse *(Mus musculus)* lines and alleles were used and interbred: *Tg(Cdh5-CreERT2)* (ref. 45), *Tg(iSuRe-Cre)* (ref. 18), *Dll1^flox/flox^* (ref. 46), *Jag1^flox/flox^* (ref. 47), *Jag2^flox/flox^* (ref. 48), *Dll4^flox/Eflox^* (ref. 49), *Notch1^fIox/flox^* (ref. 50), *Notch2^flox/flox^* (ref. 51), *Notch4^KO^* (generated as described below), *Rbpj^lox/flox^* (ref. 52), *Myc^flox/flox^* (ref. 53), *Cdkn1a(p21)^KO^* (ref. 54), *Rac1^flox/flox^* (ref. 55), *Rosa26-EYFP* (ref. 56), *iChr-Mosaic* (ref. 57) and *iMb-Mosaic* (ref. 57). To induce CreERT2 activity in adult mice, 20 mg or 10 mg of tamoxifen (Sigma-Aldrich, T5648) were first dissolved in 140 μl of absolute ethanol and then in 860 μl of corn oil (20 mg ml^-1^ or 10 mg ml^-1^ tamoxifen, respectively). From these stock solutions, dilutions were done and given to adult mice aged 2-5 months by intraperitoneal injection (total dose of 1 mg, 1.5 mg or 2 mg of tamoxifen per animal) every day for a maximum of 5 days. All mouse lines and primer sequences required to genotype these mice are provided in Supplementary Table 1.

Dll4/Notch signaling blockade in ECs was achieved using blocking antibodies to murine Dll4, developed by Regeneron (REGN1035) (ref. 58), or against Notch1 (anti-NRR1), developed by Genentech^4^. Mouse IgG (Sigma) was used in littermates as a control treatment. For the 48-h experiment, mice received a single intraperitoneal injection of 200 μl of IgG or anti-Dll4 antibody (20 mg kg^-1^ in PBS). For the 2-week blocking experiments, mice received anti-Dll4 antibody or anti-NRR1 antibody four times (day 1, day 4, day 8 and day 12) over 14 days at a concentration of 7.5 mg kg^-1^ or 10 mg kg^-1^, respectively. For anti-VEGFA treatment experiments, mouse anti-VEGFA G6-31 antibody, developed by Genentech, was administered four times over 14 days at a concentration of 5 mg kg^-1^. In mouse pups, anti-Dll4 antibody (REGN1035) was injected at 7.5 mg/kg or 20 mg/kg as indicated.

The following inhibitors were injected intraperitoneally for 2 consecutive days in postnatal animals for retina analysis, or for 4-14 consecutive days in adult animals for liver analysis as indicated in the figures. γ-secretase inhibitor DBZ (YO-01027; Selleck Chemicals, S2711) was injected at 30 μ mol kg^-1^ in adult animals every day in the morning for 4 days, and 16 h before collection of the tissues. To inhibit MAPK/ERK phosphorylation, we injected 120 mg kg^-1^ SL327 (MEK inhibitor; Selleck Chemicals, S1066) every day, and 16 h before collecting the tissues for scRNA-seq. To inhibit Rac1, we injected NSC23766 at 3 mg kg^-1^ (Sigma, SML0952). To inhibit mTOR signaling, we injected rapamycin at 4mgkg^-1^ (Enzo Life Sciences, BML-A275-0005). To inhibit NO synthase, we injected L-NIO at 30 mg kg^-1^ (R&D Systems, 0546). To inhibit Pi3K signaling, we injected alpelisib at 30 mg kg^-1^ (MedChemExpress, HY-15244).

All mouse husbandry and experimentation was conducted using protocols approved by local animal ethics committees and authorities(Comunidad Autónoma de Madrid and Universidad Autónoma de Madrid CAM-PROEX 177/14, CAM-PROEX 167/17, CAM-PROEX 164.8/20 and PROEX 293.1/22 or Uppsala Committee permit number 5.8.18-03029/2020 or the Institutional Animal Care and Use Committee Protocol IS00013945). The mouse colonies were maintained in racks with individual ventilation cages according to current national legislation. Mice had dust-free and pathogen-free bedding, and sufficient nesting and environmental enrichment material for the development of species-specific behavior. All mice had ad libitum access to food and water in environmental conditions of 45-65% relative humidity, temperatures of 21-24 °C, and a 12 h/12 h light/dark cycle. In addition, to preserve animal welfare, mouse health was monitored with an animal health surveillance program, which follows the Federation of European Laboratory Animal Science Associations (FELASA) recommendations for specific pathogen-free facilities.

We used mice with C57BL/6 or C57BL/6×129SV genetic backgrounds. To generate mice for analysis, we intercrossed mice with an age range of 7-30 weeks. Mice used for experiments were 2-5 months old. We do not expect our data to be influenced by mouse sex.

To generate *Notch4^KO^* mice, we used guide RNAs Notch4_1 (agg-gaccctcagagcccttg) and Notch4_2 (agggaatgatgccacgcata) to target mouse Notch4 in mouse eggs from the C57BL/6 genetic background. Injection mixture was composed by the described CRISPR RNA (crRNA; Integrated DNA Technologies) and trans-activating CRISPR RNA (tracrRNA; Integrated DNA Technologies, 1072533) at 0.305 μm and Cas9 nuclease (Alt-R S.p. HiFi Cas9 Nuclease V3, 100 μg, 1081060) at 20 ng μl^-1^. Founders were screened by PCR with the primers below to confirm the genetic deletion.

### Immunofluorescence on cryosections

Tissues were fixed for 2 h in 4% PFA in PBS at 4°C. After three washes in PBS for 10 min each, organs were stored overnight in 30% sucrose (Sigma) in PBS. Organs were then embedded in OCT (Sakura) and frozen at -80 °C. Cryosections (35 μm) were cut on a cryostat (Leica), washed three times for 10 min each in PBS, and blocked and permeabilized in PBS containing 10% donkey serum (Millipore), 10% fetal bovine serum (FBS) and 1% Triton X-100. Primary antibodies were diluted in blocking/ permeabilization buffer and incubated overnight at 4 °C. This step was followed by three 10-min washes in PBS and incubation for 2 h with conjugated secondary antibodies (1:200, Jackson Laboratory) and 4,6-diamidino-2-phenylindole (DAPI) in PBS at room temperature. After three washes in PBS, sections were mounted with Fluoromount- G (SouthernBiotech). All antibodies used are listed in Supplementary Table 2. To detect Ki67 or c-Myc in the same section as ERG, we used rabbit anti-Ki67 or anti-c-Myc together with a Fab fragment Cy3 secondary antibody, which is compatible with the later use of rabbit anti-ERG conjugated to Alexa Fluor 647.

### Vibratome section immunofluorescence

Tissues were fixed for 2 h in 4% PFA in PBS and washed as above. Organs were then embedded in 6% agarose low-melting gel (Invitrogen), and organ sections (100 μm) were cut on a vibratome. Sections were permeabilized for 1 h in PBS containing 1% Triton X-100 and 0.5% Tween 20. Sections were then blocked for 1h in a PBS solution containing 1% Triton X-100, 10% donkey serum and 10% FBS. Primary antibodies were diluted in blocking buffer and incubated with sections overnight at 4°C. This step was followed by six washes with 1% Triton X-100 in PBS for 15 min and incubation for 2 h with conjugated secondary antibodies (1:200,Jackson Laboratory) and DAPI in PBS at room temperature. After three 15-min washes in PBS, sections were mounted with Fluoromount-G (SouthernBiotech). All antibodies used are listed in Supplementary Table 2.

### Whole-mount immunofluorescence of retinas

For postnatal mouse retina immunostaining, eyes were collected and fixed in 4% PFA in PBS for 20 min at room temperature. After microdissection, retinas were fixed in 4% PFA for an additional 45 min, followed by two PBS washes of 10 min each. Retinas were blocked and permeabilized with PBTS buffer (0.3% Triton X-100, 3% FBS and 3% donkey serum) for 1 h. Samples were then incubated overnight at 4 °C in biotinylated isolectin B4 (diluted 1:50; Vector Laboratories, B-1205) and primary antibodies (Supplementary Table 2) diluted in PBTS buffer. After five washes of 20 min each in PBTS buffer diluted 1:2, samples were incubated for 2h at room temperature with Alexa-conjugated secondary antibodies (Thermo Fisher). After three washes of 30 min each in PBTS buffer (diluted 1:2), and two washes of 10 min each in PBS, retinas were mounted with Fluoromount-G (SouthernBiotech).

### Immunofluorescence on paraffin sections

The N1ICD epitope and the Jag1 ligand were detected with the tyramide signal amplification (TSA) kit (NEL774) procedure in paraffin sections after antigen retrieval. In brief, sections were dewaxed and rehydrated, followed by antigen retrieval in sub-boiling sodium citrate buffer (10 mM, pH 6.0) for 30 min. The slides were cooled down to room temperature for 30 min, followed by incubation for 30 min in 3% H_2_O_2_ in methanol to quench endogenous peroxidase activity. Next, slides were rinsed in double-distilled H_2_O and washed three times for 5 min each in PBS, followed by blocking for 1h in PBS containing 3% BSA, 200 mM MgCl_2_, 0.3% Tween 20 and 5% donkey serum. Sections were then incubated with primary antibody in the same solution overnight at 4 °C. After washes, slides were incubated for 2 h with anti-rabbit- HRP secondary antibody at room temperature, and, after washing, the signal was amplified using the TSA fluorescein kit (NEL774). Sections were mounted with Fluoromount-G (SouthernBiotech). All antibodies used are listed in Supplementary Table 2.

### In vivo EdU labeling and EC proliferation detection

To detect EC proliferation in adult livers, 20 μg per g body weight EdU (Invitrogen, A10044) was injected intraperitoneally into adult mice 5 h before dissection. Livers were isolated for cryosection analysis. EdU signals were detected with the Click-iT EdU Alexa Fluor 647 or 488 Imaging Kit (Invitrogen, C10340 or C10337). In brief, after all other primary and secondary antibody incubations, samples were washed according to the immunofluorescence staining procedure and then incubated with Click-iT EdU reaction cocktail for 40 min, followed by DAPI counterstaining.

### Image acquisition and analysis

Immunostained organ sections were imaged at high resolution with a Leica SP5, SP8 or SP8 Navigator confocal microscope fitted with a ×10, ×20 or ×40 objective for confocal scanning. Individual fields or tiles of large areas were acquired from cryosections, vibratome or paraffin sections. Large *Z*-volumes of the vibratome samples were imaged for 3D representation. All images shown are representative of the results obtained for each group and experiment. Animals were dissected and processed under exactly the same conditions. Comparisons of phenotypes or signal intensity were made with pictures obtained using the same laser excitation and confocal scanner detection settings. Fiji/ ImageJ was used to threshold, select and quantify objects in confocal micrographs. Both manual and automatic ImageJ public plug-ins and custom Fiji macros were used for quantification.

### Latex perfusion and CUBIC clearing

Mice were euthanized in a CO_2_ chamber. The abdominal cavity was opened, and the liver portal vein was exposed. With the help of a dissection microscope, latex (Injection Medium, Latex, Red, Laboratory Grade, Carolina, 868703) was injected in the portal vein with a 40G needle as previously described^59^. Perfusion was stopped as soon as latex was visually detectable in the liver surface vessels. Liver dissection was performed only 15 min after the perfusion to ensure latex solidification. The liver was then washed in PBS and put in PFA 4% in PBS at 4 °C overnight. After, three PBS washes for 15 min each were done at room temperature. To clear the organ, livers were incubated at 37 °C in CUBIC1 (ref. 60) solution (25 wt% urea, 25 wt% N’-Tetrakis(2- hydroxypropyl)ethylenediamine, 15 wt% Triton X-100) for a total 4 days, with the solution being exchanged every day. After clearing, liver images were captured with an Olympus camera connected to a Leica dissection scope with retroillumination. A magnification of ×0.8 was used.

### Western blot analysis

For the analysis of protein expression, livers were transferred to a reagent tube and frozen in liquid nitrogen. On the day of immunoblotting, the tissue was lysed with lysis buffer (Tris-HCl pH 8, 20 mM, EDTA 1 mM, DTT 1 mM, Triton X-100 1% and NaCl 150 mM, containing protease inhibitors (Sigma, P-8340), phosphatase inhibitors (Cal-biochem, 524629) and orthovanadate-Na 1 mM) and homogenized with a cylindrical glass pestle. Tissue and cell debris were removed by centrifugation, and the supernatant was diluted in loading buffer and analyzed by SDS-PAGE and immunoblotting. Membranes were blocked with BSA and incubated with the primary antibodies listed in Supplementary Table 2.

### EC isolation for transcriptomic and proteomic analysis

The following methods were used to isolate ECs for bulk RNA-seq, and proteomics analysis. At day 14 after the first tamoxifen injection, heart, lungs, liver and brain were dissected, minced and digested with 2.5 mg ml^-1^ collagenase type I (Thermo Fisher), 2.5 mg ml^-1^ dispase II (Thermo Fisher) and 50 ng ml^-1^ DNase I (Roche) at 37 °C for 30 min. Cells were passed through a 70-μm filter. Erythroid cells were removed by incubation with blood lysis buffer (0.15 M NH_4_Cl, 0.01 M KHCO_3_ and 0.01 M EDTA in distilled water) for 10 min on ice. Cell suspensions were blocked in blocking buffer (DPBS containing no Ca^2+^ or Mg^2+^ and supplemented with 3% dialyzed FBS; Thermo Fisher). For EC analysis, cells were incubated at 4 °C for 30 min with APC-conjugated rat anti-mouse CD31 (1:200; BD Biosciences, 551262). DAPI (5 mg ml^-1^) was added to the cells immediately before fluorescence-activated cell sorting (FACS), which was performed with FACSAria (BD Biosciences) or Synergy 4L cell sorters. For bulk RNA-seq experiments, approximately 10,000-20,000 cells for each group of DAPI-APC-CD31^+^ ECs (for Dll4 loss of function and control) and DAPI-APC-CD31^+^/MbTomato^+^ ECs (for Rbpj loss of function and control) were sorted directly to RLT buffer (RNeasy Micro Kit, Qiagen). RNA was extracted with the RNeasy Micro Kit and stored at -80 °C. For proteomic analysis, approximately 3 *10^6^ DAPI-APC-CD31^+^ ECs per group were sorted directly to blocking buffer. Cells were spun down for 10 min at 350 × *g*, and the pellet was stored at -80 °C.

To isolate ECs for scRNA-seq experiments, 1.5 mg of tamoxifen was injected on 4 consecutive days. At day 14 after the first tamoxifen injection, livers were dissected, minced and digested for 30 min with prewarmed (37 °C) dissociation buffer (2.5 mg ml^-1^ collagenase I (Thermo Fisher, 17100017), 2.5 mg ml^-1^ dispase II (Thermo Fisher, 17105041), 1 μl ml^-1^ DNase in PBS containing Ca^2+^ and Mg^2+^ (Gibco)). The digestion tube was agitated every 3-5min in a water bath. At the end of the 30-min incubation, sample tubes were filled up to 15 ml with sorting buffer (PBS containing no Ca^2+^ or Mg^2+^ and supplemented with 10% FBS (Sigma, F7524)) and centrifuged (450 ×*g*, 5 min, 4 °C). The supernatant was aspirated, and cell pellets were resuspended in 2 ml of 1x Red Blood Cell (RBC) Lysis Buffer (BioLegend, 420301) and incubated for 5 min on ice. We added 6 ml of sorting buffer to each sample, and samples were then passed through a 70-μm filter. Live cells were counted in a Neubauer chamber using trypan blue exclusion. Cells from each condition (4×10^6^ per condition) were collected in separate tubes, and cells were incubated for 30 min with horizontal rotation in 300 μl of antibody incubation buffer (PBS +1% BSA) containing 1 μl of CD31- APC, 1 μl of CD45-APC-Cy7, and 1 μl of hashtag oligo (HTO) conjugated antibodies (BioLegend). HTOs were used to label and distinguish the different samples when loaded on the same 10x Genomics port, thus also guaranteeing the absence of batch effects. After antibody incubation, samples were transferred to 15-ml Falcon tubes, 10 ml of sorting buffer were added, and samples were centrifuged (450 *×g*, 5min, 4°C). The supernatant was aspirated, pellets were resuspended in 1.5 ml of sorting buffer and transferred to Eppendorf tubes, and the resulting suspensions were centrifuged (450 ×*g*, 5 min, 4 °C). The resulting pellets were resuspended in 300 μl of sorting buffer containing DAPI. Cells were sorted with a FACSAria Cell Sorter (BD Biosciences), and CD31^+^CD45-MbTomato^+^ cells were sorted. BD FACSDiva v8.0.1 and FlowJo v10 were used for FACS data collection and analysis.

### Next-generation sequencing sample and library preparation

Next-generation sequencing experiments were performed in the Genomics Unit at Centro Nacional de Investigaciones Cardiovasculares (CNIC).

For bulk RNA-seq, control and *Dll4^iDEC^* EC samples, 1 ng of total RNA was used to amplify the cDNA using the SMART-Seq v4 Ultra Low Input RNA Kit (Clontech-Takara) following manufacturer’s instructions. Then, 1 ng of amplified cDNA was used to generate barcoded libraries using the Nextera XT DNA Library Preparation Kit (Illumina). For control and *Rbpj^iDEC^* EC samples, between 400 pg and 3,000 pg of total RNA were used to generate barcoded RNA-seq libraries using the NEBNext Single Cell/Low Input RNA Library Prep Kit for Illumina (New England Biolabs) according to manufacturer’s instructions. For control and anti-Dll4 antibody-treated ECs, libraries were generated using the Ovation Single Cell RNA-Seq System (NuGEN) following manufacturer’s instructions. All libraries were sequenced on a HiSeq 2500 (Illumina).

For scRNA-seq experiments, single cells were encapsulated into emulsion droplets using the Chromium Controller (10x Genomics). scRNA-seq libraries were prepared according to manufacturer’s instructions. The aim for target cell recovery for each port was in general 10,000 cells, with a target cell recovery of 2,000-2,500 cells per experimental condition labeled with a given hashtag antibody. Generated libraries were sequenced on a HiSeq 4000 or NextSeq 2000 (Illumina).

### Transcriptomic data analysis

Transcriptomic data were analyzed by the Bioinformatics Unit at CNIC.

For bulk RNA-seq, the number of reads per sample was between 12 million and 42 million. Reads were processed with a pipeline that assessed read quality using FastQC (Babraham Institute, http://www.bioinformatics.babraham.ac.uk/projects/fastqc) and trimmed sequencing reads using cutadapt^61^, eliminating Illumina and SMARTer adaptor remains and discarding reads with <30 base pairs (bp). More than 93% of reads were kept for all samples. The resulting reads were mapped against the mouse transcriptomes GRCm38.76 and GRCm38.91, and gene expression levels were estimated with RSEM^62^. The percentage of aligned reads was above 83% for most samples. Expression count matrices were then processed with an analysis pipeline that used Bioconductor package limma63 for normalization (using the trimmed mean *of M* values (TMM) method) and differential expression testing, taking into account only those genes expressed with at least 1 count per million (CPM) in at least two samples (the number of samples for the condition with the least replicates), and using a random variable to define blocks of samples obtained from the same animal. Changes in gene expression were considered significant if associated with a Benjamini and Hochberg-adjusted *P* value < 0.05. A complementary GSEA^64^ was performed for each contrast, using the whole collection of genes detected as expressed (12,872 genes) to identify gene sets that had a tendency to be more expressed in either of the conditions being compared. We retrieved gene sets representing pathways or functional categories from the Hallmark, Kyoto Encyclopedia of Genes and Genomes (KEGG), Reactome, and BioCarta databases, and Gene Ontology (GO) collections from the Biological Process, Molecular Function and Cellular Component ontologies from MSigDB^65^. Enriched gene sets with a false discovery rate (FDR) < 0.05% were considered of interest. Data were analyzed with Python v2.7, using the Seaborn (https://seaborn.pydata.org) and Pandas (https://pandas.pydata.org) libraries.

The following pipeline was followed for scRNA-seq data processing and in silico EC selection. For alignment and quantification of gene expression, the reference transcriptome was built using mouse genome GRCm38 and Ensembl gene build v98 (https://sep2019.archive.ensembl.org). The phiYFP-sv40pA, MbTomato-2A-Cre-WPRE-sv40pa or CreERT2 transgene sequences expressed in the samples were added to the reference. Gene metadata were obtained from the corresponding Ensembl BioMart archive. Reads from hashtags and transcripts were processed, aligned and quantified using the Cell Ranger v4.0.0 pipeline. Single-cell analysis was based on Scater^66^ and Seurat^67^ packages. Low-quality cells were filtered out using the following criteria: total counts, >1,500 and <40,000; genes detected, >600; mitochondrial transcripts content, <25%; total counts/median, >0.1; hashtag counts, >100; hemoglobin transcripts, <0.1%; and percentage of counts in the top 50 genes, <65%. Cells were demultiplexed using the sample hashtag antibody signals (BioLegend). Counts were log-normalized and scaled, followed by principal component analysis (PCA) and clustering using the shared nearest-neighbors algorithm and Louvain clustering (settings as defaults except for the 1,000 most variable genes, 10 principal components, and a resolution of 0.5). Clusters and cells were classified based on the SingleR method^68^ using Blueprint ENCODE and the Human Primary Cell Atlas cell-type profile collection. This identification was used to select ECs for the analysis and remove minor contaminants (T cells, B cells and monocytes). Hashtag-based doublets were removed, and only ECs were reclustered using the same procedure (with 2,000 variable genes, 7 PCs, a resolution of 0.3, and a random seed for uniform manifold approximation and projection (UMAP) = 123456) to get a final clustering that was later manually refined based on marker expression. Following cluster identification with the starting dataset, the remaining liver EC datasets were mapped using the FindTransfer-Anchors function from the Seurat R package using 30 PCA dimensions with the default settings.

The following pipeline was followed for liver non-EC scRNA-seq. Cells were demultiplexed by applying the cellranger multi pipeline. The following quality-control steps were performed to minimize low-quality cells and improve posterior normalization and analysis: (1) a minimum of normalized counts per cell of 2,000 and a maximum of 30,000; (2) a minimum gene detection filter of 500 genes and a maximum of 6,000; (3) a maximum mitochondria content of 5%; (4) a maximum ribosomal content of 35%; (5) a maximum hemoglobin content of 1%; and (6) only single cells were selected, and doublets were filtered out in the cellranger multi demultiplexing step. Counts were log-normalized and scaled, followed by PCA and clustering using the shared nearest-neighbors algorithm and Louvain clustering (settings as defaults except for the 2,000 most variable genes, 30 principal components and a resolution of 0.8). Clusters and cells were classified based on the SingleR method using Blueprint ENCODE, Human Primary Cell Atlas, and mouse RNA-seq datasets available in the celldex package, as well as a recent liver single-cell dataset^69^, in order to classify each cluster to a different cell type. Final clustering was later manually refined based on marker expression.

### Liver EC proteomics

Protein extraction from cell samples was carried out in the presence of SDS as described^70^. Protein concentration was determined by the RC DC Protein Assay (Bio-Rad Laboratories). Samples (100 gg) were subjected to overnight tryptic digestion using filter-aided sample preparation (FASP) technology (Expedeon)^71^. The resulting peptides were desalted on Oasis HLB C18 extraction cartridges (Waters Corporation) and dried down. The cleaned-up peptide samples were subjected to stable isotope labeling using isobaric tags for relative and absolute quantitation (iTRAQ 8-plex, AB Sciex) following the manufacturer’s instructions. The differentially tagged samples were then pooled and desalted on Oasis HLB C18 cartridges. A 100-μg aliquot of dried, labeled peptides was taken up in 0.1% trifluoroacetic acid and separated into five fractions by high pH reversed-phase peptide fractionation^72^. The bound peptides were eluted gradually with 12.5%, 15%, 17.5%, 20% and 50% acetonitrile, and the fractions obtained were vacuum-dried and stored at -20 °C for later use. The labeled peptide samples were taken up in 0.1% formic acid and analyzed on an EASY-nLC 1000 liquid chromatograph (Thermo Fisher Scientific) coupled to a Q Exactive HF mass spectrometer (Thermo Fisher Scientific). The peptide samples were loaded onto a C18 reversed-phase nano-precolumn (Acclaim PepMap 100; 75-μm internal diameter, 3-gm particle size and 2-cm length; Thermo Fisher Scientific) and separated on an analytical C18 nano-column (EASY-Spray column PepMap RSLC C18; 75-μm internal diameter, 3-μm particle size and 50-cm length; Thermo Fisher Scientific) using a linear gradient: 8-27% B for 240 min, 31-100% B for 2 min, 100% B for 7 min, 100-2% B for 2 min, and 2% B for 30 min (where A is 0.1% formic acid in high-performance liquid chromatography (HPLC)-grade water, and B is 90% acetonitrile, 0.1% formic acid in HPLC-grade water). Full MS spectra were acquired over the 400-1,500 mass-to-charge *(m/z)* range with 120,000 resolution, 2 × 10^5^ automatic gain control, and 50-ms maximum injection time. Data-dependent tandem MS (MS/ MS) acquisition was performed at 5 × 10^4^ automatic gain control and 120-ms injection time, with a 2-Da isolation window and 30-s dynamic exclusion. Higher-energy collisional dissociation of peptides was induced with 31% normalized collision energy and analyzed at 35,000 resolution in the Orbitrap. Protein identification was carried out using the SEQUEST HT algorithm integrated in Proteome Discoverer v2.1 (Thermo Fisher Scientific). MS/MS scans were matched against a mouse protein database (UniProtKB release 2017-07) supplemented with pig trypsin and human keratin sequences. Parameters for database searching were as follows: trypsin digestion with a maximum of two missed cleavage sites allowed, precursor mass tolerance of 800 ppm, and a fragment mass tolerance of 0.02 Da. Amino-terminal and Lys iTRAQ 8-plex modifications were set as fixed modifications, whereas Met oxidation, Cys carbamidomethylation, and Cys methylthiolation were set as variable modifications. The identification results were analyzed with the probability ratio method^73^. An FDR for peptide identification was calculated based on searching against the corresponding inverted database using the refined method^74,75^ after precursor mass tolerance postfiltering at 20 ppm. Quantitative information was extracted from the intensity of iTRAQ reporter ions in the low-mass region of the MS/ MS spectra^76^. The comparative analysis of protein abundance changes relied on the weighted scan peptide-protein (WSPP) statistical model^77^ by means of the SanXoT software package as described^78^. As input, WSPP uses a list of quantifications in the form of log_2_ ratios (each cell sample versus the mean of the three wild-type cell samples) with their statistical weights. From these, WSPP generates the standardized forms of the original variables by computing the quantitative values expressed in units of standard deviation around the means (Zq). For the study of coordinated protein alterations, we used the Systems Biology Triangle (SBT) algorithm, which estimates functional category averages (Zc) from protein values by performing the protein-to-category integration, as described^76^. The protein category database was built up using annotations from the GO database.

### Statistical analysis and reproducibility

All bar graphs show mean ±s.d. Experiments were repeated with independent animals. Comparisons between two groups of samples with a Gaussian distribution were by unpaired two-tailed Student’s *t*-test. Comparisons among more than two groups were made by one-way or two-way analysis of variance (ANOVA) followed by multiple comparison tests as indicated in the Source Data. All calculations were done in Microsoft Excel, and final data points were analyzed and represented with GraphPad Prism. No randomization or blinding was used, and animals or tissues were selected for analysis based on their genotype, the detected Cre-dependent recombination frequency, and the quality of multiplex immunostaining. Sample sizes were chosen according to the observed statistical variation and published protocols.

## Extended Data

## Supplementary Material

Table S1

Table S2

Supplementary Materials

## Data Availability

RNA-seq data can be viewed in the Gene Expression Omnibus (GEO) database under accession number GSE231613 (SuperSeries of GSE229793 and GSE231612). Instructions and code to reproduce all scRNA-seq results can be found at https://github.com/RuiBenedito/ Benedito_Lab. Proteomics data can be found in the Proteomics Identifications (PRIDE) database under accession number PXD041349. Unprocessed original photographs of the data are available upon request. All other data supporting the findings in this study are included in the main article and associated files.
