# Supplementary material for: Incongruence between transcriptional and vascular pathophysiological cell states
Source: Nat Cardiovasc Res. Author manuscript; Available in PMC 2023 Sep 23. (PMC7615119; doi:10.1038/s44161-023-00272-4)
Supplement: Supplementary Materials [file EMS187936-supplement-Supplementary_Materials.pdf]

nature portfolio

Corresponding author(s): Rui Benedito

Last updated by author(s): May 6, 2023

Reporting Summary

Nature Portfolio wishes to improve the reproducibility of the work that we publish. This form provides structure for consistency and transparency in reporting. For further information on Nature Portfolio policies, see our Editorial Policies and the Editorial Policy Checklist.

Statistics

For all statistical analyses, confirm that the following items are present in the figure legend, table legend, main text, or Methods section.

n/a	Confirmed	
☐	☒	The exact sample size (*n*) for each experimental group/condition, given as a discrete number and unit of measurement
☐	☒	A statement on whether measurements were taken from distinct samples or whether the same sample was measured repeatedly
☐	☒	The statistical test(s) used AND whether they are one- or two-sided<br>*Only common tests should be described solely by name; describe more complex techniques in the Methods section.*
☒	☐	A description of all covariates tested
☐	☒	A description of any assumptions or corrections, such as tests of normality and adjustment for multiple comparisons
☐	☒	A full description of the statistical parameters including central tendency (e.g. means) or other basic estimates (e.g. regression coefficient) AND variation (e.g. standard deviation) or associated estimates of uncertainty (e.g. confidence intervals)
☐	☒	For null hypothesis testing, the test statistic (e.g. *F*, *t*, *r*) with confidence intervals, effect sizes, degrees of freedom and *P* value noted<br>*Give P values as exact values whenever suitable.*
☒	☐	For Bayesian analysis, information on the choice of priors and Markov chain Monte Carlo settings
☒	☐	For hierarchical and complex designs, identification of the appropriate level for tests and full reporting of outcomes
☒	☐	Estimates of effect sizes (e.g. Cohen's *d*, Pearson's *r*), indicating how they were calculated

Our web collection on statistics for biologists contains articles on many of the points above.

Software and code

Policy information about availability of computer code

Data collection	Organ sections were imaged at high resolution with a Leica SP5, SP8, or SP8 Navigator confocal microscopes fitted with 10x, 20x, or 40x objectives for confocal scanning. Leica stereomicroscopes with Olympus Camera were used for whole liver images. Bulk RNAseq and scRNAseq data was collected with Illumina HiSeq2500, HiSeq4000 or NextSeq2000 sequencers software. BD FACS Diva V8.0.1 and Flow JO v10 was utilized for FACS data collection and analysis. Proteomic data was obtained using the SEQUEST HT algorithm integrated in Proteome Discoverer 2.1 (Thermo Fisher Scientific).

Data analysis	Numerical data was first processed with Microsoft Excel 2016 and after analysed and plotted with Graphpad Prism v7.03.<br>Microscope images were processed and analysed/quantified with ImageJ/FIJI v1.53c. Adobe Photoshop CC 19.1.5 and Adobe Illustrator CC v22.1 were used for downstream image processing, analysis and illustration.<br>Bulk RNAseq data was analysed following standard software as indicated below:<br>FastQC v0.11.5 for QC of bulk RNA-seq data.<br>RSEM v1.2.30 for quantification of gene level counts for bulk RNA-seq data.<br>limma v3.32.10 for differential gene expression analysis of bulk RNA-seq data.<br>scRNAseq was analysed following standard software as indicated below:<br>CellRanger v4.0.0. for processing, alignment, and quantification of reads from hashtags and transcripts.<br>Seurat package for de-multiplexing, normalization and counts analysis.<br>Transcriptional data analysis with Python 2.7, using the Seaborn (https://seaborn.pydata.org) and Pandas libraries (https://pandas.pydata.org/).<br>Proteomics data was analysed following standard software as indicate below:<br>SanXoT software package for comparative analysis of protein abundance changes.

For manuscripts utilizing custom algorithms or software that are central to the research but not yet described in published literature, software must be made available to editors and reviewers. We strongly encourage code deposition in a community repository (e.g. GitHub). See the Nature Portfolio guidelines for submitting code & software for further information.

Data

Policy information about availability of data

All manuscripts must include a data availability statement. This statement should provide the following information, where applicable:

- Accession codes, unique identifiers, or web links for publicly available datasets
- A description of any restrictions on data availability
- For clinical datasets or third party data, please ensure that the statement adheres to our policy

RNA-seq data can be viewed in the Gene Expression Omnibus (GEO) database under accession number GSE231613 (SuperSeries of GSE229793 and GSE231612). Instructions and code to reproduce all scRNA-seq results can be found at https://github.com/RuiBenedito/Benedito_Lab. Proteomics data can be found in the Proteomics Identifications (PRIDE) database under accession number PXD041349 . Unprocessed original photographs of the data are available upon request. All other data supporting the findings in this study are included in the main article and associated files.

Human research participants

Policy information about studies involving human research participants and Sex and Gender in Research.

Reporting on sex and gender	N/A
Population characteristics	N/A
Recruitment	N/A
Ethics oversight	N/A

Note that full information on the approval of the study protocol must also be provided in the manuscript.

Field-specific reporting

Please select the one below that is the best fit for your research. If you are not sure, read the appropriate sections before making your selection.

☒ Life sciences ☐ Behavioural & social sciences ☐ Ecological, evolutionary & environmental sciences

For a reference copy of the document with all sections, see nature.com/documents/nr-reporting-summary-flat.pdf

Life sciences study design

All studies must disclose on these points even when the disclosure is negative.

Sample size	Sample size was determined taking into account the 3 R´s for animal experimentation and the expected experimental variability based on published and our own protocols (i.e. Luo et al., 2021, Inverso et al., 2021 and Winkler et al., 2021). The final sample size was defined at posteriori based on the statistical analysis of the data. Sample size and related statistical analysis methods are indicated in the manuscript methods section or source data file 1.<br>Luo, W. et al. Arterialization requires the timely suppression of cell growth. Nature 589, 437-441, doi:10.1038/s41586-020-3018-x (2021).<br>Inverso, D. et al. A spatial vascular transcriptomic, proteomic, and phosphoproteomic atlas unveils an angiocrine Tie-Wnt signaling axis in the liver. Dev Cell 56, 1677-1693 e1610, doi:10.1016/j.devcel.2021.05.001 (2021).

	Winkler, M. et al. Endothelial GATA4 controls liver fibrosis and regeneration by preventing a pathogenic switch in angiocrine signaling. J Hepatol 74, 380-393, doi:10.1016/j.jhep.2020.08.033 (2021).
Data exclusions	Data was excluded only if technical problems were detected. These include technical problems detected after immunostaining and microscopy analysis or insufficient reporter recombination or gene deletion in experiments involving CreERT2 alleles and conditional genetics.
Replication	Data shown in charts are the mean of independent biological repeats. The n number of animals used for each comparison is stated in the source data file 1. Experiments were repeated several times with different animals to guarantee maximum reproducibility. In addition, the main conclusions are supported by several different experiments using different techniques (transcriptomis, proteomics and phenotypic analysis).
Randomization	Animals/tissues were selected for a posteriori analysis based on their genotype, the detected Cre-dependent recombination frequency, and quality of multiplex immunostaining.
Blinding	Investigators were not blinded during data collection or analysis due to its impracticality and need for a priori knowledge of which control and mutant samples are being handled and selected for analysis, so that all downstream costs and analysis are kept to the minimum necessary. ImageJ/FIJI software was used to analyse the microscopy data in an automatic and objective manner. All experiments in the paper were quantified utilizing standardized experimental controls and quantitative methods to avoid bias.

Reporting for specific materials, systems and methods

We require information from authors about some types of materials, experimental systems and methods used in many studies. Here, indicate whether each material, system or method listed is relevant to your study. If you are not sure if a list item applies to your research, read the appropriate section before selecting a response.

Materials & experimental systems

n/a	Involved in the study	
☐	☒	Antibodies
☒	☐	Eukaryotic cell lines
☒	☐	Palaeontology and archaeology
☐	☒	Animals and other organisms
☒	☐	Clinical data
☒	☐	Dual use research of concern

Methods

n/a	Involved in the study	
☒	☐	ChIP-seq
☐	☒	Flow cytometry
☒	☐	MRI-based neuroimaging

Antibodies

Antibodies used

All detailed info is contained in Supplementary Table 2.
Anti-GFP/YFP/Cerulean 1:200 (IF) Acris Antibodies Cat# R1091P
Anti-DsRed 1:400 (IF) Clontech Cat# 632496
Anti-HA -647 1:200 (IF) Cell Signaling Technology Cat# 3444S
Anti-ERG 1:400 (IF) Abcam Cat# ab110639
Anti-ERG-AF-647 1:200 (IF) Abcam Cat# ab196149
Anti-Ki67 1:200 (IF) Thermo Fisher Cat# RM-9106-S0
Anti-Ki67-e660 1:200 (IF) Thermo Fisher Cat# 50-5698-82
Anti-Endomucin 1:200 (IF) Santa Cruz Biotechnology Cat# SC-53941
Anti-CD31 1:200 (IF) BD Biosciences Cat# 553370
Anti-CD31 1:200 (FC) BD Biosciences Cat# 740680
Anti-p21 1:10 (IF) CNIO (now at Abcam) Cat# HUGO291
Anti-p21 1:100 (IF) Santa Cruz Biotechnology Cat# SC-397-G
Anti-p42/44 (Total ERK) 1:1000 (WB) Cell Signaling Technology Cat# 9102
Anti-p42/44-Phospho-ERK 1:100 (IF) Cell Signaling Technology Cat# 4370S
1 to 1000 (WB)
Anti-Myc 1:200 (IF) Millipore Cat# 06-340
Anti-cleaved N1ICD 1:200 (IF) Cell Signaling Technology Cat# 4147
Anti-Dll4 1:200 (IF) R&D system Cat# AF1389
Anti-Jagged1 1: 100 (IF) Cell Signaling Technology Cat# 2620
Anti-CD34-FITC 1:200 (IF) BD Biosciences Cat# 560238
Anti-CD68 1:200 (IF) Bio-Rad MCA1957
Anti-CD45 1:200 (IF) BD Biosciences Cat# 550539
Anti-CD45 1:200 (FC) TonboBio Cat# 35-0454-U100
Anti-Caspase 3 1:50 (IF) Cell Signaling Technology Cat# 9661S
Anti-Esm1 1:200 (IF) R&D system Cat# AF1999
Anti-Msr1 1:200 (IF) R&D system Cat# AF1797-SP
Anti-CD11b 1:200 (FC) BD Biosciences Cat# 561690
Anti-Ly6C 1:200 (FC) BD Biosciences Cat# 561085
Anti-Ly6G 1:200 (FC) Biolegend Cat# 123113
Donkey Anti-Goat 488 1:400 (IF) Thermo Fisher Cat # A-11055

Donkey Anti-Goat 633 1:400 (IF) Thermo Fisher Cat # A-21082
Donkey Anti-Rabbit 594 1:400 (IF) Jackson Immunoresearch Cat # 711-587-003
Donkey Anti-Rabbit 488 1:400 (IF) Jackson Immunoresearch Cat # 711-547-003
Donkey Anti-Rabbit 647 1:400 (IF) Jackson Immunoresearch Cat # 711-607-003
Donkey Anti-Rat 488 1:400 (IF) Thermo Fisher Cat # A-21208
Donkey Anti-Rat 647 1:400 (IF) Abcam Cat # ab150155

Validation

All antibodies used are commercially available and have been pre-validated by the companies and us. They all gave immunostaining or immunoblotting results according to what was expected from their previously published tissue expression pattern, subcellular localization or the predicted immunoblot target protein size.

Animals and other research organisms

Policy information about studies involving animals; ARRIVE guidelines recommended for reporting animal research, and Sex and Gender in Research

Laboratory animals

We used Mus musculus with C57BL6 or C57BL6×129SV genetic backgrounds. To generate male and female mice for analysis, we intercrossed mice with an age range between 7 and 30 weeks. Mice used for experiments had between 2 and 5 months. We do not expect our data to be influenced by mouse sex or age.
All mouse husbandry and experimentation was conducted using protocols approved by local animal ethics committees and authorities (Comunidad Autónoma de Madrid and Universidad Autónoma de Madrid CAM-PROEX 177/14, CAM-PROEX 167/17, CAM-PROEX 164.8/20 and PROEX 293.1/22 or Uppsala Committee permit number 5.8.18-03029/2020 or the Institutional Animal Care and Use Committee Protocol IS00013945). The mouse colonies (Mus musculus) were maintained in racks with individual ventilation cages according to current national legislation. Mice have dust/pathogen-free bedding, and sufficient nesting and environmental enrichment material for the development of species-specific behavior. All mice have 'ad libitum' access to food and water in environmental conditions of 45–65% relative humidity, temperatures of 21–24°C, and a 12 h/12 h light/dark cycle. In addition, and to preserve animal welfare, mouse health is monitored with an animal health surveillance program, which follows FELASA recommendations for specific pathogen-free facilities.Details about the transgenic or gene-targeted allleles used are provided in the Methods section, under Mice.

Wild animals

No wild animals were used in the study.

Reporting on sex

We do not expect our data to be influenced by animal age or sex.

Field-collected samples

No field collected samples were used in the study.

Ethics oversight

All mouse husbandry and experimentation was conducted using protocols approved by local animal ethics committees and authorities (Comunidad Autónoma de Madrid and Universidad Autónoma de Madrid CAM-PROEX 177/14, CAM-PROEX 167/17, CAM-PROEX 164.8/20 and PROEX 293.1/22 or Uppsala Committee permit number 5.8.18-03029/2020 or the Institutional Animal Care and Use Committee Protocol IS00013945).

Note that full information on the approval of the study protocol must also be provided in the manuscript.

Flow Cytometry

Plots

Confirm that:

☒ The axis labels state the marker and fluorochrome used (e.g. CD4-FITC).

☒ The axis scales are clearly visible. Include numbers along axes only for bottom left plot of group (a 'group' is an analysis of identical markers).

☒ All plots are contour plots with outliers or pseudocolor plots.

☒ A numerical value for number of cells or percentage (with statistics) is provided.

Methodology

Sample preparation

We used FACS plots to obtain numeric data or to isolate endothelial cells from mouse tissues. We included these FACS plots and the gating strategy used in Extended Data Figures.
The following methods were used to isolate ECs for bulk RNA-seq, and proteomics analysis. At day 14 after the first tamoxifen injection, heart, lungs, liver, and brain were dissected, minced and digested with 2.5 mg/ml collagenase type I (Thermofisher), 2.5 mg/ml dispase II (Thermofisher), and 50 ng/ml DNAseI (Roche) at 37°C for 30 min. Cells were passed through a 70 µm filter. Erythroid cells were removed by incubation with blood lysis buffer (0.15 M NH_4Cl, 0.01M $KHCO_3$, and 0.01 M EDTA in distilled water) for 10 min on ice. Cell suspensions were blocked in blocking buffer (DPBS containing no Ca^{2+} or Mg^{2+} and supplemented with 3% dialyzed FBS; Thermo Fisher). For EC analysis, cells were incubated at 4°C for 30 min with APC-conjugated rat anti-mouse CD31 (1:200, BD Bioscience, 551262). DAPI (5 mg/ml) was added to the cells immediately before FACS, which was performed with FACS Aria (BD Biosciences) or Synergy4L cell sorters. For bulk RNA-seq experiments, approximately 10000-20000 cells for each group of DAPI-negative APC-CD31+ ECs (for Dll4 loss of function and control), DAPI negative APC-CD31+/MbTomato+ ECs (for Rbpj loss of function and control) were sorted directly to RLT buffer (RNAeasy Micro kit - Qiagen). RNA was extracted with the RNAeasy Micro kit and stored at -80°C. For proteomic analysis,

approximately 3x106 DAPI-negative APC-CD31+ ECs per group were sorted directly to blocking buffer. Cells were spun down for 10 min at 350g and pellet stored at -80°C.
To isolate ECs for scRNA-seq experiments, 1.5 mg tamoxifen was injected on 4 consecutive days. At day 14 after the first tamoxifen injection, livers were dissected, minced, and digested for 30 min with pre-warmed (37C) dissociation buffer (2.5mg/ml collagenase I - Thermo Fisher 17100017), 2.5mg/ml dispase II (Thermo Fisher 17105041), 1ul/ml DNAse in PBS containing Ca2+ and Mg2+ (Gibco)). The digestion tube was agitated every 3-5 minutes in a water bath. At the end of the 30 minutes incubation, sample tubes were filled up to 15 ml with sorting buffer (PBS containing no Ca2+ or Mg2+ and supplemented with 10% FBS (Sigma, F7524)) and centrifuged (450g, 5 min, 4°C). The supernatant was aspirated, and cell pellets were resuspended in 2ml 1x RBC lysis buffer (BioLegend, 420301) and incubated for 5 min on ice. To each sample were added 6 ml of sorting buffer, and samples were then passed through a 70um filter. Live cells were counted in a Neubauer Chamber using trypan blue exclusion. Cells from each condition (4x106/condition) were collected in separate tubes, and cells were incubated for 30 min with horizontal rotation in 300µl antibody incubation buffer (PBS + 1% BSA) containing 1 µl CD31-APC, 1 µl CD45-APC-Cy7, and 1µl of hash tag oligo (HTO) conjugated antibodies (Biolegend). HTOs were used to label and distinguish the different samples when loaded on the same 10x genomics port, and in this way also guarantee the absence of batch effects. After the antibodies incubation, samples were transferred to 15 ml Falcon tubes, 10 ml sorting buffer was added, and samples were centrifuged (450g, 5min, 4°C). The supernatant was aspirated, pellets were resuspended in 1.5 ml sorting buffer and transferred to Eppendorf tubes, and the resulting suspensions were centrifuged (450g, 5min, 4°C). The resulting pellets were resuspended in 300 µl sorting buffer containing DAPI. Cells were sorted by FACS with an Aria Cell Sorter (BD Biosciences) and CD31+, CD45- MbTomato+ cells were sorted. BD FACS Diva V8.0.1 and Flow JO v10 was utilized for FACS data collection and analysis.

Instrument

BD FACS ARIA

Software

BD FACS Diva V8.0.1

Cell population abundance

For each group, approximately 10000-20000 DAPI negative APC-CD31+ cells without or with fluorescence (MbTomato) were sorted to individual tubes. For proteomic analysis, 3000000 endothelial cells were sorted in each sample. The purity of the samples was very high given the endothelial profile of the Transcriptomic and Protemic analysis.

Gating strategy

The gating strategy is indicated in Extended Data Fig. 1 and 4.

☒ Tick this box to confirm that a figure exemplifying the gating strategy is provided in the Supplementary Information.
